# DNA Methylation of Pharmacologic and Leukemia-Related Genes Predicts Clinical Outcomes in Pediatric Acute Myeloid Leukemia

**DOI:** 10.3390/cancers18152467

**Published:** 2026-07-31

**Authors:** Naifah Alshameri, Francisco Marchi, Xueyuan Cao, Jeffrey E. Rubnitz, Raul C. Ribeiro, Soheil Meshinchi, Stanley B. Pounds, Jatinder K. Lamba

**Affiliations:** 1 Department of Pharmacotherapy and Translational Research, College of Pharmacy, University of Florida, Gainesville, FL 32610, USA; 2 University of Hail, Hail 81451, Saudi Arabia; 3 Department of Health Promotion/Disease Prevention, University of Tennessee Health Science Center, Memphis, TN 38163, USA; xcao12@uthsc.edu; 4 Ascentage Pharma Group, Rockville, MD 20850, USA; jeff.rubnitz@ascentage.com; 5 Department of Oncology, St. Jude Children’s Research Hospital, Memphis, TN 38105, USA; 6 Division of Hematology/Oncology, Fred Hutchinson Cancer Research Center, Seattle, WA 98109, USA; 7 Department of Biostatistics, St. Jude Children’s Research Hospital, Memphis, TN 38105, USA; 8 UF Health Cancer Center, University of Florida, Gainesville, FL 32610, USA

**Keywords:** acute myeloid leukemia, DNA methylation survival

## Abstract

Background: Aberrant DNA methylation is a hallmark of acute myeloid leukemia (AML) and contributes to leukemogenesis, treatment response, and clinical heterogeneity. While genome-wide methylation studies have identified prognostic methylation signatures, the impact of DNA methylation within pharmacologic pathways and AML-relevant disease genes remains incompletely understood. We investigated the association of DNA methylation in genes of pharmacokinetic/pharmacodynamic (PK/PD) pathways of drugs used to treat AML and in myeloid leukemia-related genes with treatment outcomes in pediatric AML. Methods: DNA methylation profiles from 924 pediatric AML patients treated on Children’s Oncology Group trials (AAML1031, AAML0531, and AAML03P1—available publicly) were analyzed as a discovery cohort. A validation cohort included 159 patients treated on the AML02 trial. A total of 2296 variable CpG sites mapping to 65 PK/PD genes and 107 AML biology/leukemia stemness genes were evaluated. Associations between CpG methylation, gene expression, event-free survival (EFS), overall survival (OS), and measurable residual disease after induction I (MRD1) were assessed using Cox proportional hazards, logistic regression, and correlation analyses. Results: Twenty-three CpG sites in PK/PD genes and forty-two CpG sites in AML-related genes were significantly associated with at least one clinical endpoint after Bonferroni correction (*p* < 2.17 × 10^−5^). Hypermethylation of drug transporters *ABCA3*, *ABCC1*, and *SLC22A1*, as well as pharmacologically relevant genes *MPO*, *NOS3*, and *CTPS1*, was associated with inferior survival and/or increased MRD1 positivity. Among AML biology genes, methylation of *ETV6*, *NOTCH1*, *RUNX1*, *KIT*, *MPL*, *DNMT3A*, and *DNMT3B* demonstrated consistent associations with outcomes across discovery and validation cohorts. Several genes exhibited significant inverse correlations between DNA methylation and gene expression, including *MPO*, *KIT*, *MPL*, *SPINK2*, and *DNMT3B*, supporting functional epigenetic regulation. Notably, hypermethylation of *ABCA3*, *MPO*, and *MPL* was reproducibly associated with poor OS and EFS in both cohorts. Conclusions: DNA methylation of key pharmacologic and leukemia-related genes is associated with clinical outcomes in pediatric AML. These findings identify biologically and clinically relevant epigenetic biomarkers that may improve risk stratification and support the development of precision medicine approaches incorporating DNA methylation profiling and epigenetic therapies in pediatric AML.

## Simple Summary

Acute myeloid leukemia is an aggressive hematological cancer with highly uncontrolled blast cell clonal expansion and proliferation. The poor prognosis of AML is an major ongoing concern; thus, studying the possible mechanisms that lead to AML progression or chemotherapy resistance may define new biomarkers and therapeutic targets that will improve diagnosis and predictions. The primary objective of this study was to evaluate the impact of the methylation levels of specific genes and their association with treatment outcomes among pediatric AML patients. Here, we demonstrated that interpatient differences in DNA methylation levels of key genes related to AML drugs or disease pathogenesis affect clinical outcomes. This creates opportunities for outcome prediction and the strategic use of hypomethylating agents to enhance patient clinical outcomes.

## 1. Introduction

Acute myeloid leukemia (AML) is characterized by highly proliferative nonfunctional cells. AML represents 15–20% of leukemia cases in the US and is the primary cause of leukemia-related deaths in children [1,2]. The five-year overall survival (OS) rate is around 70%, and the 5-year event-free survival (EFS) rate is around 65% for pediatric AML. Despite an initial response, a significant proportion of patients experience relapse or resistant disease [3,4,5]. Since the 1970s, the “7 + 3 chemotherapy regimen” (7 days of cytarabine and 3 days of daunorubicin) has been used for treating AML patients, with cytarabine, daunorubicin and etoposide being the commonly used induction regimen in children [6]. However, 20% of pediatric AML patients experience relapse after standard induction chemotherapy [7]. Treatment failure, because of refractory or relapsed disease, remains the major cause of death in AML patients. In-depth understanding of the molecular mechanism underlying disease pathology and drug resistance is thus needed. Multiple studies have shown the importance of aberrant DNA methylation in the onset of leukemia [8,9,10]. Previous reports have shown that 29.7% of adult patients with AML have DNA methylation-regulated gene mutations, which are further associated with inferior clinical outcomes. Our previous work in pediatric AML showed that distinctive DNA methylation signatures are associated with specific cytogenetic features. An integrated analysis of DNA methylation and gene expression revealed *DNMT3B* methylation and expression as well as global DNA methylation burden to be of clinical and prognostic relevance in pediatric AML [11].

Despite the fact that multiple studies have investigated DNA methylation profiles in AML, a systematic investigation of how methylation patterns of genes involved in pharmacokinetic and pharmacodynamic pathways of standard chemotherapeutic agents such as cytarabine, daunorubicin, and etoposide contribute to clinical outcome and drug resistance is lacking. Furthermore, in-depth investigation of methylation patterns of genes of pathological relevance in myeloid leukemia and their impact on clinical outcome is needed. Thus, this study evaluated associations between methylation of PK/PD genes and a panel of genes relevant to AML pathology with clinical outcomes in pediatric AML patients.

## 2. Methods

**Patient Samples:** The discovery cohort included 924 pediatric AML patients treated on Children’s Oncology Group clinical trials AAML1031, AML0531 and AML03P1 where the DNA methylation and clinical outcome data are available in a public database (see below). The median age at diagnosis for the patients was 9.7 years (interquartile range (IQR) 3.1–14.8 years). The studies were approved by the National Cancer Institute’s central institutional review board (IRB) and the IRB at each participating centre. Written informed consent was obtained from participants or their caregivers. The validation cohort consisted of 159 patients treated on the AML02 trial (NCT00136084) with DNA methylation and clinical outcome data available as per previous reports [11,12,13]. Median age at diagnosis was 9.3 years (IQR: 2.2, 14.0). Details of the clinical trials are described elsewhere [7,14,15,16].

The clinical outcomes endpoints were EFS, defined from the period from study entry until the first occurrence of death, refractory disease, or any type of relapse with event-free patients censored on the date of last follow-up; OS, defined as the period from study entry to the time of death with living patients censored on the date of last follow-up; and minimal residual disease after induction 1 (MRD1), with positivity defined as 1 or more leukemic cells per 1000 mononuclear bone marrow cells (≥0.1%) determined using flow cytometry.

**Target Gene List:** Geneset-1 (PK/PD Pathway Genes): We selected 65 genes based on their relevance to the pharmacological pathways of cytarabine, daunorubicin, and etoposide. The PharmGKB database and literature reports were used to extract the genes of relevance [17,18].

Geneset-2 (Myeloid and Leukemia Stemness Genes): We selected 107 genes that are implicated in AML pathogenesis and genes associated with leukemia stem cells (LSCs) from a previously reported study [19,20]. Details are provided in the Supplementary Methods and the gene list is provided in Supplementary Table S1.

**DNA Methylation Data:** The DNA methylation raw data were obtained from publicly available datasets from Gene Expression Omnibus (GEO) (AAML1031-GSE190931, AAML0531-GSE124413) processed by Illumina Human Methylation EPIC array, and Genomic Data Commons (AAML03P1-GDC-TARGET AML) processed by Illumina Human Methylation 450K array. The EPIC and 450K arrays share 452,453 probes with the same chemistry and design. Details regarding DNA isolation, bisulfite conversion, and hybridization of bisulfite-converted DNA have been reported in GEO and GDC for the discovery cohort [21,22,23], and the validation cohort AML02 has been previously published [12]. The methylation array 450k/EPIC array data were pre-processed using the R package *SeSAMe* as described in the Supplementary Methods. Methylation levels of CpGs mapping to both gene sets represented on both arrays were extracted. CpG sites were filtered for variability across the cohort. Only those with a standard deviation greater than 0.01 were included in the analysis, resulting in a total of 2296 CpGs.

**Gene Expression Data:** For the discovery cohort COGs, the expression levels of the pre-selected genes were extracted from RNA-seq data, performed on bone marrow samples obtained at diagnosis, using the Illumina HiSeq2000 platform from the Therapeutically Applicable Research to Generate Effective Treatments (TARGET) database. To integrate the RNA-seq data from three datasets and adjust for related batch effects, we used ComBat in R software. All RNA-seq values were log2-normalized (log2(RPKM +1)) for all subsequent analyses. Of the 924 patients from the discovery cohort, 745 samples had available clinical data and gene expression data. For the validation cohort AML02, the mRNA expression levels in diagnostic leukemic blasts from patient samples were obtained using Affymetrix U133A microarray data as previously described [24]. Gene expression values were transformed using the natural logarithm before data analysis. One hundred and thirty-six patients in the validation cohort had DNA methylation and gene expression data available for further downstream analysis.

**Statistical Analysis:** R software version 4.2.3 was used to conduct the statistical analysis. Pearson’s Chi-squared test and Welch’s two-sample *t*-test were utilized to summarize baseline patient characteristics for both discovery and validation cohorts. The Kruskal–Wallis test was performed to test the difference in DNA methylation level of CpGs among risk groups and race groups. For DNA methylation analysis, M values were evaluated for association with OS and EFS using the *Survival* package [25]. Cox regression models were used to calculate hazard ratios (HRs) and 95% confidence intervals (CIs) for the association of DNA methylation levels (M-values) with OS and EFS. The risk-adjusted Cox model is as follows:

$$h_{i}\left(t\right)=h_{0}\left(t\right)\times \exp\;\{\beta 1M_{i}+\beta 2R_{i,\mathrm{Standard}}+\beta 3R_{i,\mathrm{High}}\}$$

where $h_{i}\left(t\right)\;$ represents the hazard for patient i at time t and $h_{0}\left(t\right)$ represents the baseline hazard. $M_{i}\;$denotes the methylation level. $R_{i,\mathrm{Standard}}\;$ and $R_{i,\mathrm{High}}\;$ are indicator variables for the standard- and high-risk groups, with the low-risk group as a reference category. $\exp\left(\beta_{1}\right)$ represents the ratio associated with the methylation level after adjustment for clinical risk group. Bonferroni correction was applied to adjust for multiple testing with the alpha level set to ***α*** = 2.17 × 10^−5^. Survival curves were plotted using the Kaplan–Meier method and compared using the log-rank test, stratified by the median of M values. Logistic regression was applied to assess the association between CpG methylation M value and MRD1 status (positive or negative). The *ggplot2* package was used to create MRD1 bar plots for the significant CpG sites based on the median of M values [26]. The CpGs associated with clinical outcomes were further evaluated for association with respective gene expression. Spearman correlation analysis was used to evaluate the relationship between gene expression and DNA methylation (5mC).

## 3. Results

The baseline characteristics of the patients in the study cohort are summarized in Table 1.

**Figure 1:**
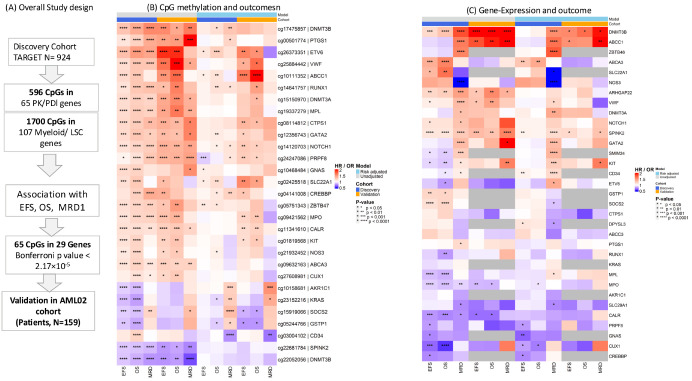
(**A**) Overall strategy of the work. (**B**) Heatmap showing association (unadjusted and risk-adjusted analysis) of top selected CpGs with EFS, OS and MRD1 in discovery and validation cohorts. (**C**) Heatmap showing association (unadjusted and risk-adjusted analysis) of gene expression of candidate genes with EFS, OS and MRD1 in discovery and validation cohorts. The density of the heatmap reflects the hazard ratio for EFS and OS and odds ratio for MRD1. A significant *p*-value is reflected by an asterisk, and grey boxes in the gene expression heatmap reflect missing data in the validation cohort.

**Association of DNA Methylation of PK/PD with Clinical Outcome:** Twenty three of the 596 CpGs tested in drug pathway genes and 42 of the 1700 CpGs mapping to genes associated with AML disease biology showed significant association with at least one clinical endpoint at the Bonferroni-corrected *p*-value threshold of <2.17 × 10^−5^ (summarized in Supplementary Table S2). Figure 1 shows a heatmap of the most significant CpG selected for each of these genes for association with clinical outcome in the discovery and validation cohorts (both unadjusted and risk-adjusted; detailed results are provided in Table 2). Additionally, association of respective mRNA expression levels of genes with available data with outcomes is also shown in Figure 1C.

**Figure 2:**
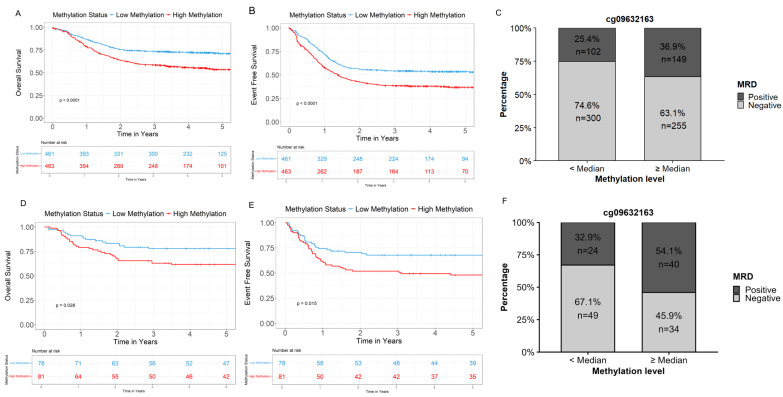
Association of methylation levels of an intronic Cg09632163 in ABCA3 with outcome in pediatric AML. Kaplan–Meier survival curves of median methylation values for OS and EFS in the discovery cohort (**A**,**B**) and validation cohort (**D**,**E**). Stacked bar plot showing the proportion of those who were MRD-negative (light grey) or MRD-positive (dark grey) by ABCA3 methylation (by median) in discovery and validation cohorts (**C**,**F**).

Among drug transporters, *ABCA3*, *ABCC1* and *SLC22A1* showed significant associations with clinical outcome endpoints. Methylation levels of seven intronic CpGs in efflux transporter ABCA3 were associated with clinical outcome endpoints in both discovery and validation cohorts. The methylation levels of all seven CPGs were correlated with each other (Supplementary Figure S1). Of these, for the most significant CpG (cg09632163), greater methylation was associated with poor OS (HR = 1.23, 95% CI 1.16–1.30, *p* < 10^−12^), poor EFS (HR = 1.14, 95% CI 1.10–1.19, *p* < 10^−10^), and higher MRD1 positivity (OR = 1.29, *p* < 0.0031, T) in the discovery cohort. In the validation cohort, consistent results were observed with cg09632163 associated with inferior OS (HR = 1.22, 95% CI 1.05–1.42, *p* = 0.0089) and EFS (HR = 1.25, 95% CI 1.099–1.415, *p* = 0.0006), and higher MRD1 positivity (OR = 1.23, 95% CI 1.066–1.438, *p* = 0.006) (Figure 2). In the risk-adjusted analysis, *ABCA3* was not associated with any of the outcomes, which was in part due to a significant association of *ABCA3* methylation with cytogenetic risk groups with the highest methylation in high-risk-group patients (*p* = 4.61 × 10^−32^, Figure S2). Further, the higher *ABCA3* expression was associated with worse OS (HR = 1.62, 95% CI 1.31–2.02, *p* < 10^−5^) and EFS (HR = 1.42, 95% CI 1.18–1.72, *p* = 0.0002) in the discovery cohort (Table 3 and Supplementary Figures S3 and S4). No significant association with ABCA3 expression was observed in the validation cohort.

Hypermethylation of cg10111352 in the intronic region of another efflux transporter, *ABCC1*, was associated with poor OS (HR = 1.29, 95% CI 1.18–1.40, *p* < 2.42 × 10^−9^) and EFS (HR = 1.2, 95% CI 1.12–1.29, *p* < 1.7 × 10^−7^); consistent results were seen in the AML02 validation cohort with worse OS (HR = 1.74, 95% CI 1.34–2.27, *p* = 4.22 × 10^−5^) and EFS (HR = 1.54, 95% CI 1.24–1.92, *p* = 0.0001). In risk-adjusted analysis, *ABCC1* methylation was significantly associated with EFS and OS in both discovery and validation cohorts (heatmap, Figure 1). For organic anion uptake transporter *SLC22A1*, higher methylation was associated with poor EFS and OS in discovery and validation cohorts (Figure 1 and Supplementary Table S2).

**Figure 3:**
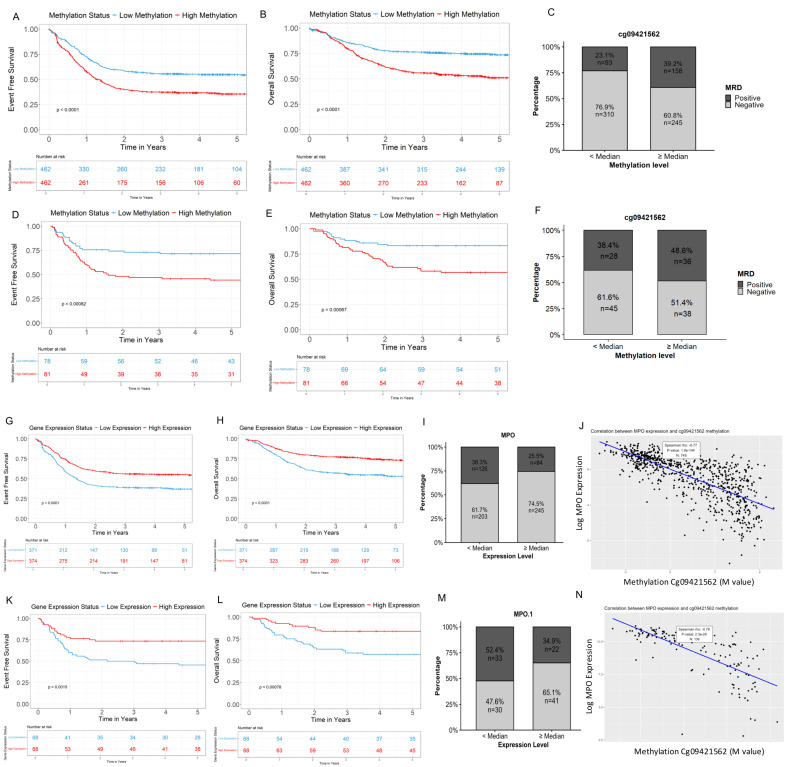
Association of methylation levels of an intronic Cg09421562 in MPO with outcome in pediatric AML. Kaplan–Meier survival curves of median methylation values for OS and EFS in the discovery cohort (**A**,**B**) and validation cohort (**D**,**E**). Stacked bar plot showing the proportion of those who were MRD-negative (light grey) or MRD-positive (dark grey) by MPO methylation (by median) in discovery and validation cohorts (**C**,**F**). Association of expression levels of MPO with outcome in pediatric AML. Kaplan–Meier survival curves of median expression values for OS and EFS in the discovery cohort (**G**,**H**) and validation cohort (**K**,**L**). Stacked bar plot showing the proportion of those who were MRD-negative (light grey) or MRD-positive (dark grey) by MPO expression (by median) in discovery and validation cohorts (**I**,**J**). The correlation between MPO Cg09421562 methylation levels and gene expression is shown in scatter plots for discovery (**M**) and validation cohorts (**N**).

Myeloperoxidase (MPO) is a myeloid marker gene which also plays a role in etoposide pharmacology. Hypermethylation at eight CpGs on the *MPO* locus was significantly associated with adverse clinical outcome. The methylation levels of all eight CPGs were significantly correlated with each other (Supplementary Figure S1). Among them, cg09421562 was the most significant CpG with hypermethylation associated with poor OS in discovery (HR = 1.22, 95% CI 1.15–1.29, *p* < 7.63 × 10^−11^), EFS (HR = 1.15, 95% CI 1.10–1.20, *p* < 8.37 × 10^−9^) and MRD1 (OR = 1.23, *p* < 9.5 × 10^−7^, Table 2) and validation cohorts (OS: HR = 1.38, 95% CI 1.173–1.636, *p* < 0.0001; EFS: HR = 1.32, 95% CI 1.155–1.51, *p* < 4.55 × 10^−5^) (Figure 3). Hypermethylation of all CpGs in the MPO gene including cg09421562 was significantly associated with lower MPO gene expression (ρ = −0.77, *p* < 10^−10^, Figure 3J,N). Consistent with this, increased MPO expression was related to better clinical outcomes in discovery (OS: HR = 0.87, 95% CI 0.83–0.90, *p* < 10^−11^; EFS: HR = 0.89, 95% CI 0.86–0.93, *p* < 10^−9^) and validation cohorts (OS: HR = 0.84, 95% CI 0.73–0.97, *p* = 0.014; EFS: HR = 0.85, 95% CI 0.75–0.96, *p* = 0.007) (Figure 3G–M). Hypermethylation of CpG (cg21932452) located in the intron of nitric oxide synthase 3 (NOS3) was significantly associated with inferior outcome in both discovery and validation cohorts (discovery cohort: OS: HR = 1.21, 95% CI 1.13–1.30, *p* < 10^−8^; EFS: HR = 1.12, 95% CI 1.06–1.19, *p* < 0.0001; validation cohort: OS: *p* = 0.02 and EFS: *p* = 0.03). For CTPS1, a CTP synthase involved in Ara-C activation, higher methylation was associated poor OS (HR: 1.25, 95% CI 1.13–1.4, *p* = 1.75 × 10^−5^), EFS (HR: 1.19, 95% CI 1.09–1.34, *p* = 9.25 × 10^−5^) and MRD1 (OR 1.355, 95% CI 1.14–1.618, *p* = 0.001) in the discovery cohort and with poor OS (*p* = 0.004), EFS (*p* = 0.0004) and MRD1 (*p* = 0.034) in the validation cohort (Table 2 and Figure 1).

**Figure 4:**
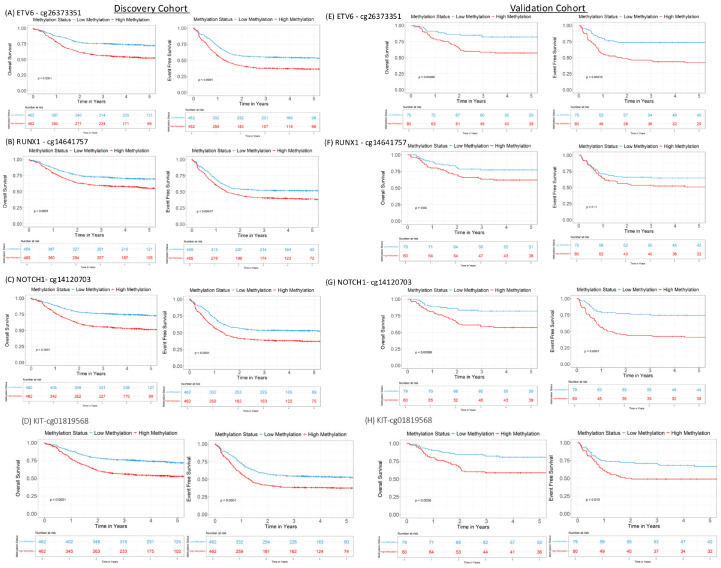
Association of methylation levels of ETV6—cg26373351, RUNX1—cg14641757, NOTCH1- cg14120703 and KIT-cgg01819568 with OS and EFS in discovery and validation cohorts. Kaplan–Meier survival curves of median methylation values for OS and EFS in the discovery cohort (**A**–**D**) and validation cohort (**E**–**H**).

**Association of DNA Methylation of AML Genes with Clinical Outcome**: Among genes of relevance to myeloid malignancies, *ETV6* (two CpGs), *NOTCH1* (three CpGs), *RUNX1* (one CpG) and *KIT* (five CpGs), significant association between higher methylation and poor OS, EFS and MRD1 was observed in discovery and validation cohorts (Figure 1 and Figure 4). Additionally favourable OS and EFS outcomes were observed among patients with high KIT expression (OS: HR = 0.88, 95% CI 0.81–0.96, *p* = 0.003; EFS: HR = 0.91, 95% CI 0.85–0.98, *p* = 0.009) in the discovery cohort; such an association was not observed in the validation cohort (Figure S5). A significant inverse correlation was observed between the methylation and expression levels of *KIT* (ρ  = −0.45, *p* < 10^−7^; Figures S3 and S5). For SPINK2, a gene previously reported to be part of a leukemic stemness signature, higher methylation was associated with better OS, EFS and MRD1 in both discovery (OS: HR = 0.87, *p* = 3.05 × 10^−6^; EFS: HR = 0.88, *p* = 1.19 × 10^−7^; MRD1: OR = 0.85, *p* = 7.34 × 10^−5^) and validation cohorts (OS: HR = 0.81, *p* = 0.015; EFS: HR = 0.78, *p* = 0.001; MRD1: OR = 0.74, *p* = 0.001; Table 2 and Figure S6) and its higher expression was associated with poor outcome in both discovery (OS: HR = 1.13, *p* = 7.08 × 10^−6^; EFS: HR = 1.13, *p* = 5.55 × 10^−8^; MRD1: OR, 1.27, *p* = 2 × 10^−10^) and validation cohorts (OS: HR = 1.31, *p* = 0.004; EFS: HR = 1.35, *p* = 0.002; MRD1: OR = 1.63, *p* = 2 × 10^−5^) (Table 3). Methylation levels of cg22681784 and SPINK2 expression were significantly negatively correlated in both discovery and validation cohorts (*p* < 10 × 10^−6^).

**Figure 5:**
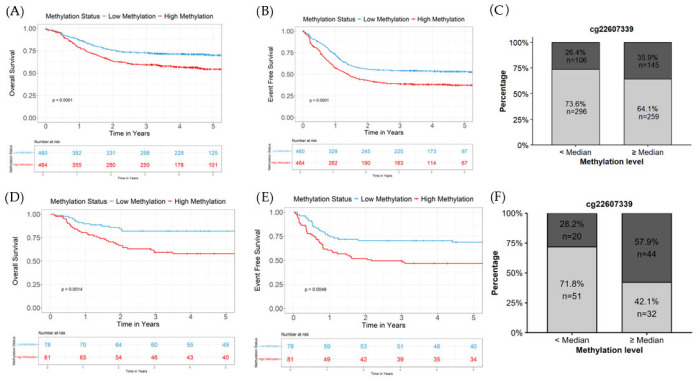
Association of methylation levels of an intronic Cg22607339 in MPL with outcome in pediatric AML. Kaplan–Meier survival curves of median methylation values for OS and EFS in the discovery cohort (**A**,**B**) and validation cohort (**D**,**E**). Stacked bar plot showing the proportion of those who were MRD-negative (light grey) or MRD-positive (dark grey) by MPL methylation (by median) in discovery and validation cohorts (**C**,**F**). Association of expression levels of MPL with outcome in pediatric AML.

We observed that an increase in DNA methylation level of cg22607339 in Myeloproliferative Leukemia Virus Oncogene (MPL) was significantly associated with poor outcome in discovery (OS: HR = 1.18, 95% CI 1.10–1.27, *p* < 10^−6^; EFS: HR = 1.14, 95% CI 1.07–1.21, *p* < 10^−5^; MRD1: OR = 1.19, *p* = 0.001) and validation cohorts (Figure 5). Further expression and methylation of MPL showed negative correlation (ρ  = −0.51, *p* < 10^−10^; Supplementary Figure S7) and as expected, higher MPL expression was significantly associated with better survival outcome (OS: HR = 0.83, 95% CI 0.76–0.91, *p* < 10^−5^; EFS: HR = 0.86, 95% CI 0.80–0.93, *p* < 10^−5^) in the discovery cohort.

Six CpGs in DNMT3A and three intronic CpGs in DNMT3B showed hypermethylation to be significantly associated with poor EFS, OS and MRD1 in both discovery and validation cohorts (Table 2 and Table S2; Figure S8 shows one of the DNMT3B CpGs). For the promoter CpG in *DNMT3B*, cg22052056, higher expression was associated with better EFS, OS and MRD1. Methylation of *DNMT3B* was significantly associated with its expression and in risk-adjusted analysis, *DNMT3B* intronic CpGs stayed significant in their association with the clinical endpoints. Among other genes, *AKR1C1*, *KRAS*, *SOCS2*, *GSTP1* and CD34, we observed significant association only in the discovery cohort, and risk-adjusted analysis showed inconsistent association with outcome endpoints.

In a multivariable analysis adjusting for risk group, age and race (Supplementary Table S3), methylation levels of CpGs in *ABCC1*, *ETV6*, *KIT, NOS3*, *NOTCH1*, and *RUNX1* were associated with OS in PRPF8, and stayed independent predictors of OS and/or EFS. Some of the associations were lost in the multivariable analysis, as those with ABCC1 and CpGs in *MPO*, *DNMT3B*, *VWF* and *CALR* stayed significant predictors of OS and EFS only in the validation cohort.

## 4. Discussion

AML is an aggressive and biologically heterogeneous malignancy associated with poor clinical outcomes. Aberrant epigenetic regulation, particularly altered DNA methylation, has been implicated in AML pathogenesis, and increased DNA methylation has been associated with inferior survival in both adult and pediatric AML [27]. Despite numerous studies investigating genome-wide methylation patterns in AML, no previous study has evaluated the prognostic significance of DNA methylation in genes involved in the pharmacology of AML therapeutics alongside key genes implicated in AML biology. In this study we report results from systematic investigation of DNA methylation of both drug-pharmacology and AML disease-pathway genes with treatment outcomes in pediatric AML.

Notably, the findings involving drug transporters (*ABCA3*, *ABCC1* and *SLC22A1*) provide novel insight into mechanisms of treatment resistance in AML [28]. ABC transporters have long been implicated in multidrug resistance through altered intracellular drug accumulation. Our observations suggest that epigenetic dysregulation of transporter genes may represent an additional mechanism contributing to variability in chemotherapy response. *ABCA3* has been previously associated with failure to achieve remission in patients and, in in vitro studies, with resistance to multiple drugs including doxorubicin [29,30]. In particular, the consistent association of *ABCA3* methylation with adverse outcomes across independent cohorts highlights its potential clinical relevance. Interestingly, although higher *ABCA3* methylation and higher *ABCA3* expression were both associated with inferior outcomes, the significant association between *ABCA3* methylation and adverse-risk cytogenetics suggests that these CpG markers may reflect broader epigenetic programmes associated with aggressive disease biology rather than direct transcriptional repression alone. *ABCC1* has been shown to limit the efficacy of venetoclax in AML [31,32]. Additional functional studies are warranted to clarify these relationships.

Among other pharmacologically relevant genes, DNA methylation levels of *CTPS1*, *NOS3* and *MPO* were associated with outcomes. *CTPS1* is involved in cytarabine metabolism and nucleotide biosynthesis, while NOS3 has been implicated in anthracycline response and toxicity. MPO plays an important role in the oxidative activation of etoposide and has long been recognized as a diagnostic marker of AML due to its selective expression in myeloid cells. Previous studies have demonstrated an inverse relationship between MPO methylation and expression in newly diagnosed AML patients, and treatment with hypomethylating agents has been shown to restore MPO expression [33]. Furthermore, MPO-mediated generation of reactive oxygen species and nitrated tyrosine residues has been linked to chemotherapy-induced apoptosis, suggesting a potential mechanistic connection between *MPO* methylation, reduced expression, and treatment resistance [34].

Among genes involved in AML biology, in addition to previously reported *DNMT3A*, *DNMT3B,* significant methylation–outcome associations were observed for *ETV6*, *KIT*, *NOTCH1*, *RUNX1* and *MPL*; for all these genes, DNA methylation was also associated with corresponding gene expression. These genes play critical roles in hematopoietic development, stem cell maintenance, and leukemic transformation. For example, *KIT*, a receptor tyrosine kinase mutated in 4–6% of newly diagnosed AML cases, has established roles in leukemic cell proliferation and survival, while *RUNX1* and *ETV6* are essential regulators of hematopoietic stem and progenitor cell function.

*MPL* (thrombopoietin receptor) is of particular interest as it is critical for hematopoietic stem cell maintenance and self-renewal and has been implicated in hematologic disorders characterized by cytopenias and progression to AML [35]. We observed that increased *MPL* methylation was associated with inferior clinical outcomes and reduced gene expression, suggesting that epigenetic silencing of *MPL* may contribute to impaired hematopoietic regulation and adverse prognosis. Similarly, methylation-associated suppression of genes involved in stem cell regulation and hematopoietic differentiation may contribute to persistence of treatment-resistant leukemic populations.

One of the important clinical implications of these findings is their potential utility for improving risk stratification. Current AML risk classification is driven primarily by cytogenetic and molecular abnormalities along with MRD1 assessment. Despite these advances, substantial heterogeneity in treatment response and survival remains within established risk groups. The methylation markers identified in this study may provide complementary prognostic information beyond conventional risk factors and warrant future evaluation as components of integrated risk prediction models. Such approaches could help identify patients at elevated risk of treatment failure who may benefit from alternative therapeutic strategies or intensified monitoring.

Because DNA methylation is a key epigenetic regulator of gene expression, we examined the relationship between CpG methylation and transcript abundance. We identified significant correlations between DNA methylation at specific CpG sites and the expression of their corresponding genes. Although promoter methylation-mediated gene silencing is well established, Yang et al. demonstrated that gene body hypomethylation can also reduce gene expression, highlighting the complex regulatory role of DNA methylation and its potential as a therapeutic target in cancer [36].

Consistent with these observations, we found positive correlations between methylation of CpG sites within the *ABCA3* gene body and *ABCA3* expression. In contrast, higher methylation levels within the gene bodies of *MPO* and *KIT* were associated with lower gene expression. The functional role of gene body methylation remains incompletely understood. Proposed mechanisms include transcriptional repression through chromatin condensation and interactions with regulatory elements such as enhancers, transcription factor binding sites, and repetitive elements. Conversely, accumulating evidence suggests that gene body methylation can promote gene expression by regulating alternative splicing and maintaining transcriptional fidelity [37]. Our findings further indicate that the relationship between DNA methylation and gene expression is highly context-dependent. Specifically, increased methylation within the *DNMT3B* promoter was associated with reduced *DNMT3B* expression, whereas methylation within the gene body showed the opposite association (Figure S8). Together, these findings underscore the complexity of DNA methylation-mediated gene regulation and highlight the importance of CpG genomic context in determining transcriptional outcomes, warranting further mechanistic investigation.

The therapeutic implications of these findings are particularly relevant given the increasing incorporation of hypomethylating agents, including azacitidine and decitabine, into AML treatment regimens. Initially approved for myelodysplastic syndromes, these agents have become integral components of AML therapy, particularly for older or medically unfit patients. In 2018, the FDA approved venetoclax in combination with azacitidine or decitabine for adults with newly diagnosed AML who are ineligible for intensive chemotherapy, and in 2020, oral azacitidine was approved as maintenance therapy for patients who achieved remission but were unable to complete intensive curative treatment [38,39,40]. The rationale underlying these agents is reversal of aberrant DNA methylation and restoration of transcriptional activity. Consequently, the pharmacologic and leukemia-associated genes identified in our analysis represent biologically plausible and potentially actionable epigenetic targets. The inverse relationship observed between DNA methylation and gene expression for several genes, including *MPO*, *KIT*, *MPL*, *SPINK2***,** and *DNMT3B,* further supports the functional relevance of these epigenetic alterations. Future studies should evaluate whether specific methylation signatures can identify patients most likely to benefit from epigenetic therapies or rational combination approaches incorporating hypomethylating agents.

This study has several limitations. First, its retrospective design limits causal inference. Second, gene expression was measured using different platforms in the discovery and validation cohorts, which may have contributed to the differences observed in multivariable analyses. Finally, DNA methylation was assessed in bulk leukemic samples and therefore may not fully capture the cellular heterogeneity of AML or the epigenetic landscape of rare subpopulations, such as leukemia stem cells.

In summary, we identified DNA methylation markers within both pharmacologic and AML disease-related genes that are associated with treatment response and survival in pediatric AML. These findings provide new insight into the epigenetic mechanisms underlying chemotherapy resistance and leukemia biology while highlighting the potential clinical utility of DNA methylation biomarkers for risk stratification and therapeutic decision-making. Prospective validation and mechanistic studies are warranted to establish the predictive value of these methylation markers and to determine their potential as therapeutic targets, particularly in the context of emerging epigenetic treatment strategies.

## Figures and Tables

**Table 1:** Baseline demographics and characteristics of patients included in this study.

Characteristic	Discovery, n = 924	Validation, n = 159
Age in Years, Median (IQR)	9.7 (3.1, 14.8)	9.3 (2.2, 14.0)
Female Sex	457 (49%)	74 (47%)
Primary Cytogenetics		
Inv (16)	111 (12%)	19 (12%)
11q23 Rearrangements	188 (21%)	35 (22%)
Normal Karyotype	217 (24%)	40 (25%)
t(8;21)	129 (14%)	19 (12%)
Other	259 (29%)	46 (29%)
Race		
White	680 (79%)	114 (72%)
Black or African American	100 (12%)	26 (16%)
Other	81 (9.4%)	18 (11%)
Risk Group		
High Risk	126 (14%)	46 (29%)
Low Risk	341 (37%)	49 (31%)
Standard Risk	443 (49%)	64 (40%)

IQR, interquartile range; Inv (16), inversion in chromosome 16; t(8;21), translocation between chromosomes 8 and 21. Values are presented as numbers (percentages) unless otherwise noted.

**Table 2:** Association of DNA methylation of selected genes with OS and EFS in the discovery cohort and validation cohort.

			**OS Discovery (n = 924)**				**EFS Discovery (n = 924)**				**MRD Discovery (n = 924)**			
**Probe ID**	**CpG_Region**	**Gene**	**HR**	**Lower 95% CI**	**Upper 95% CI**	***p*** **-Value ^1^**	**HR**	**Lower 95% CI**	**Upper 95% CI**	***p*** **-Value ^1^**	**OR**	**Lower 95% CI**	**Upper 95% CI**	***p*** **-Value ^1^**
cg09632163	Intron	ABCA3	1.23	1.162	1.303	1.30 × 10^−12^	1.143	1.096	1.191	3.63 × 10^−10^	1.247	1.099	1.415	0.001
cg10111352	Intron	ABCC1	1.286	1.184	1.397	2.42 × 10^−9^	1.2	1.121	1.285	1.70 × 10^−7^	1.541	1.236	1.92	1.2 × 10^−4^
cg09421562	Intron	MPO	1.218	1.148	1.292	7.68 × 10^−11^	1.148	1.096	1.204	8.37 × 10^−9^	1.32	1.155	1.509	4.6 × 10^−5^
cg21932452	Intron	NOS3	1.209	1.128	1.295	6.33 × 10^−8^	1.12	1.056	1.186	1.00 × 10^−4^	1.21	1.017	1.44	0.032
cg00501774	Promoter	PTGS1	1.237	1.123	1.363	1.69 × 10^−5^	1.138	1.052	1.231	1.30 × 10^−3^	1.469	1.136	1.901	0.003
cg11341610	Promoter	CALR	1.172	1.105	1.244	1.61 × 10^−7^	1.165	1.108	1.224	2.24 × 10^−9^	1.248	1.12	1.391	0.000
cg15150970	Promoter	DNMT3A	1.248	1.15	1.355	1.18 × 10^−7^	1.181	1.106	1.262	6.84 × 10^−7^	1.446	1.164	1.795	0.001
cg26373351	Intron	ETV6	1.523	1.344	1.726	4.32 × 10^−11^	1.33	1.205	1.469	1.60 × 10^−8^	1.94	1.395	2.697	0.000
cg01819568	Intron	KIT	1.218	1.141	1.3	2.92 × 10^−9^	1.147	1.089	1.21	3.14 × 10^−7^	1.233	1.068	1.423	0.004
cg19337279	Exon	MPL	1.259	1.152	1.376	3.85 × 10^−7^	1.186	1.11	1.267	4.43 × 10^−7^	1.456	1.142	1.855	0.002
cg14120703	Intron	NOTCH1	1.218	1.15	1.291	2.17 × 10^−11^	1.159	1.104	1.216	2.41 × 10^−9^	1.403	1.217	1.618	0.000
cg24247086	Promoter	PRPF8	1.156	1.085	1.231	7.59 × 10^−6^	1.057	1.008	1.108	2.20 × 10^−2^	1.387	1.198	1.605	0.000
cg14641757	Intron	RUNX1	1.288	1.168	1.421	3.79 × 10^−7^	1.154	1.071	1.244	2.00 × 10^−4^	1.339	1.062	1.688	0.013
cg17475857	Intron	DNMT3B	1.434	1.283	1.603	2.19 × 10^−10^	1.301	1.187	1.426	1.84 × 10^−8^	1.591	1.21	2.092	0.001
cg22052056	Promoter	DNMT3B	0.831	0.775	0.891	2.04 × 10^−7^	0.876	0.826	0.928	7.16 × 10^−6^	0.765	0.663	0.884	0.000
cg12356743	Promoter	GATA2	1.294	1.165	1.437	1.51 × 10^−6^	1.195	1.083	1.32	4.00 × 10^−4^	1.355	1.12	1.64	0.002
cg25884442	Intron	VWF	1.296	1.179	1.425	7.84 × 10^−8^	1.178	1.094	1.267	1.22 × 10^−5^	1.699	1.251	2.308	0.001
cg08114812	Exon	CTPS1	1.255	1.132	1.393	1.75 × 10^−5^	1.193	1.0918	1.304	9.52 × 10^−5^	1.416	1.168	1.718	0.0004
cg22681784	Intron	SPINK2	0.869	0.82	0.922	3.05 × 10^−6^	0.88	0.8398	0.923	1.19 × 10^−7^	0.778	0.67	0.903	0.001
cg05751343	Intron	ZBTB47	1.215	1.117	1.322	0.0000057	1.174	1.099	1.254	1.94 × 10^−6^	1.301	1.06	1.598	0.012
cg04141008	Promoter	CREBBP	1.241	1.127	1.367	0.000012	1.134	1.0494	1.226	1.226	1.42	1.133	1.78	0.002
cg08114812	Exon	CTPS1	1.255	1.132	1.393	1.75 × 10^−5^	1.193	1.0918	1.304	9.52 × 10^−5^	1.416	1.168	1.718	0.0004
cg27608981	Intron	CUX1	1.156	1.087	1.23	3.80 × 10^−6^	1.079	1.028	1.133	1.133	1.204	1.046	1.386	0.01
			**OS Validation (n = 159)**				**EFS Validation (n = 159)**				**MRD Validation (n = 159)**			
**Probe ID**	**CpG_Region**	**Gene**	**HR**	**Lower 95% CI**	**Upper 95% CI**	***p*** **-Value**	**HR**	**Lower 95% CI**	**Upper 95% CI**	***p*** **-Value**	**OR**	**Lower 95% CI**	**Upper 95% CI**	***p*** **-Value**
cg09632163	Intron	ABCA3	1.134	1.061	1.217	0.0003	1.223	1.052	1.423	0.0089	1.232	1.066	1.438	0.0060
cg10111352	Intron	ABCC1	1.091	0.966	1.232	0.1600	1.743	1.336	2.274	4.22 × 10^−5^	1.043	0.767	1.418	0.7860
cg09421562	Intron	MPO	1.23	1.133	1.337	9.53 × 10^−7^	1.386	1.173	1.636	0.0001	1.103	0.929	1.312	0.2650
cg21932452	Intron	NOS3	1.009	0.911	1.116	0.8620	1.279	1.038	1.576	0.0208	1.019	0.8	1.298	0.8800
cg00501774	Promoter	PTGS1	1.433	1.244	1.655	0.0000	1.511	1.122	2.035	0.0066	2.166	1.479	3.29	0.0001
cg11341610	Promoter	CALR	1.155	1.058	1.262	0.0013	1.277	1.127	1.447	0.0001	1.213	1.02	1.454	0.0320
cg15150970	Promoter	DNMT3A	1.221	1.086	1.375	0.0009	1.589	1.221	2.067	0.0006	1.403	1.052	1.899	0.0240
cg26373351	Intron	ETV6	1.464	1.239	1.739	1.05 × 10^−5^	1.942	1.318	2.862	0.0008	1.3	0.866	1.978	0.2110
cg01819568	Intron	KIT	1.05	0.962	1.147	0.2740	1.305	1.094	1.558	0.0031	1.09	0.906	1.315	0.3620
cg19337279	Exon	MPL	1.235	1.107	1.386	0.0002	1.679	1.196	2.357	0.0028	1.415	1.111	1.876	0.0090
cg14120703	Intron	NOTCH1	1.257	1.151	1.374	3.69 × 10^−7^	1.389	1.174	1.644	0.0001	1.454	1.155	1.861	0.0020
cg24247086	Promoter	PRPF8	1.287	1.176	1.413	7.13 × 10^−8^	1.404	1.173	1.681	0.0002	1.474	1.23	1.795	0.0001
cg14641757	Intron	RUNX1	1.32	1.16	1.509	3.36 × 10^−5^	1.641	1.184	2.274	0.0029	1.311	1.004	1.76	0.0570
cg17475857	Intron	DNMT3B	1.595	1.358	1.881	1.91 × 10^−8^	1.45	1.069	1.969	0.0171	1.648	1.148	2.427	0.0090
cg22052056	Promoter	DNMT3B	0.771	0.696	0.853	4.78 × 10^−7^	0.782	0.663	0.923	0.0037	0.614	0.479	0.774	0.0001
cg12356743	Promoter	GATA2	1.252	1.042	1.505	0.0200	1.334	1.063	1.673	0.0128	1.622	1.1	2.614	0.0250
cg25884442	Intron	VWF	1.3	1.146	1.479	0.0001	2.13	1.402	3.237	0.0004	1.445	1.043	2.053	0.0320
cg08114812	Exon	CTPS1	1.355	1.142	1.618	0.001	1.362	1.102	1.102	0.0043	1.637	1.087	2.774	0.035
cg22681784	Intron	SPINK2	0.85	0.783	0.92	7.3376 × 10^−5^	0.807	0.679	0.959	0.015	0.736	0.605	0.884	0.001
cg05751343	Intron	ZBTB47	1.243	1.111	1.396	0.0002	1.521	1.158	1.999	0.0026	1.259	0.98	1.646	0.079
cg04141008	Promoter	CREBBP	1.423	1.243	1.636	4.4499 × 10^−7^	1.294	0.998	1.678	0.0515	1.134	0.873	1.488	0.352
cg08114812	Exon	CTPS1	1.355	1.142	1.618	0.001	1.362	1.102	1.102	0.0043	1.637	1.087	2.774	0.035
cg27608981	Intron	CUX1	1.094	1.005	1.19	0.038	1.273	1.072	1.511	0.0058	1.124	0.941	1.349	0.199

OS, Overall Survival; EFS, Event-Free Survival; HR, Hazard Ratio; CI, Confidence Interval. ^1^ Bonferroni-corrected alpha level α = 2.1777 × 10^−5^ for discovery.

**Table 3:** Association of gene expression of candidate genes with OS, EFS and MRD in discovery and validation cohorts in pediatric AML patients.

	**OS Discovery (n = 745)**				**EFS Discovery (n = 745)**				**MRD1 Discovery (n = 658)**			
**Gene**	**HR**	**Lower 95% CI**	**Upper 95% CI**	***p*** **-Value**	**HR**	**Lower 95% CI**	**Upper 95% CI**	***p*** **-Value**	**OR**	**Lower 95% CI**	**Upper 95% CI**	***p*** **-Value**
ABCA3	1.625	1.306	2.022	1.32 × 10^−5^	1.422	1.178	1.716	0.0002	1.116	1.116	1.548	0.515
ABCC1	0.889	0.761	1.038	0.135	0.917	0.809	1.04	0.178	1.664	1.664	2.087	7.3 × 10^−6^
MPO	0.866	0.83	0.904	4.61 × 10^−11^	0.896	0.864	0.929	2.41 × 10^−9^	0.905	0.905	0.966	0.003
CALR	0.738	0.619	0.879	0.0007	0.773	0.671	0.89	0.0004	0.757	0.757	0.96	0.022
ETV6	0.779	0.639	0.949	0.013	0.886	0.753	1.043	0.147	1.294	1.294	1.704	0.063
KIT	0.879	0.807	0.958	0.003	0.911	0.849	0.978	0.009	1.135	1.135	1.28	0.038
MPL	0.829	0.755	0.91	8.76 × 10^−5^	0.86	0.798	0.926	7.23 × 10^−5^	1.024	1.024	1.151	0.692
NOTCH1	1.154	0.983	1.354	0.08	1.152	1.011	1.314	0.034	1.305	1.305	1.617	0.014
PRPF8	0.895	0.694	1.153	0.39	0.782	0.637	0.96	0.019	0.763	0.763	1.053	0.01
RUNX1	0.779	0.663	0.915	0.002	0.903	0.79	1.031	0.132	0.93	0.93	1.154	0.509
DNMT3B	1.311	1.168	1.472	4.19 × 10^−6^	1.193	1.085	1.31	0.0002	1.843	1.843	2.19	1.6 × 10^−12^
CUX1	0.564	0.432	0.736	2.44 × 10^−5^	0.673	0.539	0.839	0.0004	0.96	0.96	1.389	0.826
VWF	1.099	0.996	1.214	0.061	1.087	1.003	1.178	0.042	1.355	1.355	1.558	1.8 × 10^−5^
SPINK2	1.129	1.071	1.19	7.08 × 10^−6^	1.127	1.08	1.177	5.55 × 10^−8^	1.273	1.273	1.373	1.6 × 10^−10^
	**OS Validation (n = 132)**				**EFS Validation (n = 132)**				**MRD Validation (n = 126)**			
**Gene**	**HR**	**Lower 95% CI**	**Upper 95% CI**	***p*** **-Value**	**HR**	**Lower 95% CI**	**Upper 95% CI**	***p*** **-Value**	**OR**	**Lower 95% CI**	**Upper 95% CI**	***p*** **-Value**
ABCA3	0.88	0.575	1.34	0.546	0.827	0.578	1.182	0.298	1.119	0.701	1.795	0.638
ABCC1	1.97	1.187	3.283	0.009	1.838	1.204	2.808	0.005	3.674	1.947	7.496	0.0001
MPO	0.84	0.732	0.966	0.014	0.846	0.749	0.955	0.007	1.015	0.813	1.271	0.898
CALR	0.69	0.481	0.985	0.041	0.691	0.513	0.93	0.015	0.87	0.552	1.359	0.54
ETV6	0.67	0.381	1.181	0.166	0.793	0.497	1.265	0.33	0.645	0.333	1.215	0.18
KIT	1.11	0.844	1.465	0.449	1.189	0.937	1.509	0.155	1.634	1.146	2.43	0.01
MPL	0.97	0.61	1.552	0.909	0.855	0.563	1.299	0.464	0.779	0.534	1.12	0.183
NOTCH1	1.27	0.683	2.347	0.454	1.412	0.8	2.492	0.234	1.832	0.924	3.803	0.091
PRPF8	0.62	0.285	1.358	0.233	0.97	0.455	2.069	0.938	0.656	0.206	1.941	0.449
RUNX1	0.96	0.705	1.314	0.808	0.963	0.739	1.254	0.777	0.821	0.598	1.117	0.212
DNMT3B	2.28	1.569	3.305	1.51 × 10^−5^	2.043	1.49	2.801	9.13 × 10^−6^	3.377	2.061	5.892	5 × 10^−6^
CUX1	0.75	0.348	1.601	0.453	0.755	0.398	1.431	0.389	3.292	1.127	10.44	0.035
VWF	1.48	1.151	1.892	0.002	1.312	1.055	1.631	0.014	1.262	0.899	1.79	0.182
SPINK2	1.31	1.091	1.57	0.004	1.352	1.152	1.587	0.0002	1.631	1.315	2.068	2 × 10^−5^

## Data Availability

The data used in this study is publicly available through the TARGET database.

## Supplementary Materials

The following supporting information can be downloaded at: https://www.mdpi.com/article/10.3390/cancers18152467/s1, Figure S1. Correlation Heatmap showing association of CpG methylation for the genes with multiple CpGs associated with outcome in discovery cohort. Figure S2. Box plots showing association of DNA methylation levels of individual genes by risk groups. Figure S3. Heatmap showing association gene expression with CpG methylation (discovery- RNAseq and validation cohort-U133A array) of selected CpGs that were associated with clinical outcome in Figure 1. Top bar shows the cohort (blue- discovery and orange validation cohort). p values are shown by * and direction of correlation blue negative and red positive. Grey indicates data not available in validation cohort. Figure S4. Association of expression levels of ABCA3 with outcome in pediatric AML. Kaplan-Meier survival curves for by median expression values for OS and EFS in discovery cohort (A and B) and validation cohort (D and E). Correlation between ABCA3 Cg09632163 methylation levels and gene expression is shown in scatter plots for discovery (C) and validation cohorts (F). Figure S5. Association of methylation levels of a KIT expression with outcome in pediatric AML. Kaplan-Meier survival curves for by median methylation values for OS and EFS in discovery cohort (A and B) and validation cohort (D and E). Correlation between MPO Cg09421562 methylation levels and gene expression is shown in scatter plots for discovery (C) and validation cohorts (F). Figure S6. Association of methylation levels (cg22681784) and Gene Expression SPINK2 with outcome in pediatric AML. Kaplan-Meier survival curves for by median methylation values (A–C) and SPINK2 Expression (D–F) for OS and EFS and MRD1 in discovery cohort and validation cohort (G–L). Stacked bar plot showing the proportion of who were MRD negative (light grey), or MRD positive (dark grey) by MPO methylation (by median) in discovery and validation cohorts (C and F in discovery and I and L in validation cohort). Figure S7. Association of MPL expression with low expression with OS (A) and EFS (B) in discovery cohort. (C) Scatterplot showing correlation of MPL methylation with expression in discovery cohort. Figure S8. Association of methylation levels of an intronic Cg17475857 and Cg22052056 in DNMT3B with outcome in pediatric AML. Kaplan-Meier survival curves for by median methylation values for OS and EFS in discovery cohort (A and B) for Cg17475857 and (E and F) for Cg22052056. Stacked bar plot showing the proportion of who were MRD negative (light grey), or MRD positive (dark grey) and Correlation between DNMT3B Cg17475857 and Cg22052056 methylation levels with gene expression is shown in scatter plots (D and H). Table S1: List of CpGs and genes investigated in this study. Table S2. Association of Methylation of CpGs in candidate genes with clinical outcome (Unadjusted and Risk Adjusted Analysis) in discovery and validation cohorts.
