# Supplementary material for: DNA Methylation of Pharmacologic and Leukemia-Related Genes Predicts Clinical Outcomes in Pediatric Acute Myeloid Leukemia
Source: Cancers (Basel). 2026 Jul 31;18(15):2467. doi: 10.3390/cancers18152467 (PMC13465556; doi:10.3390/cancers18152467)
Supplement: Supplementary file 1 [file cancers-18-02467-s001.zip › cancers-4424876-supplementary.pdf]

SUPPLEMENTARY FIGURES

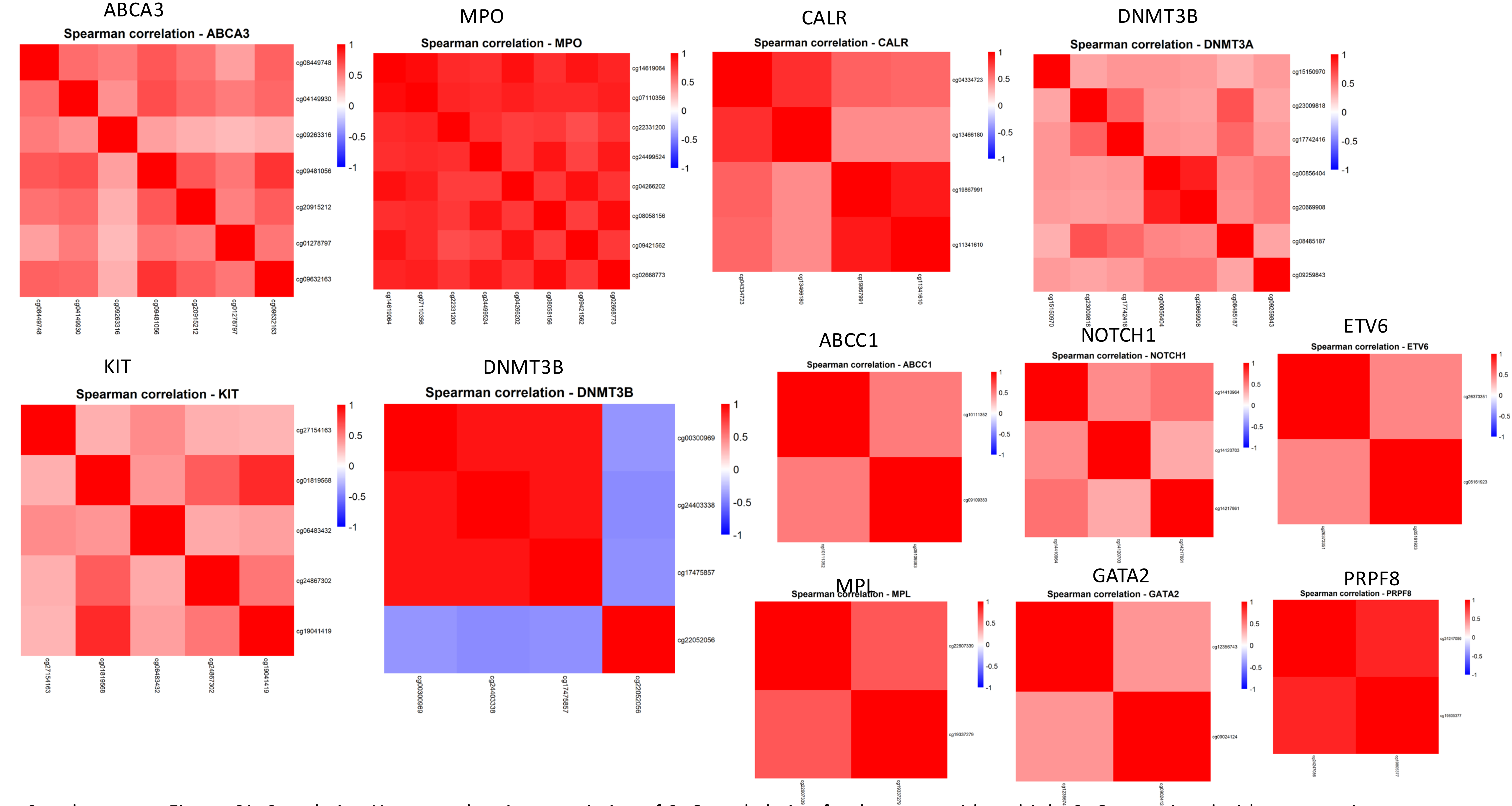

Supplementary Figure S1. Correlation Heatmap showing association of CpG methylation for the genes with multiple CpGs associated with outcome in discovery cohort.

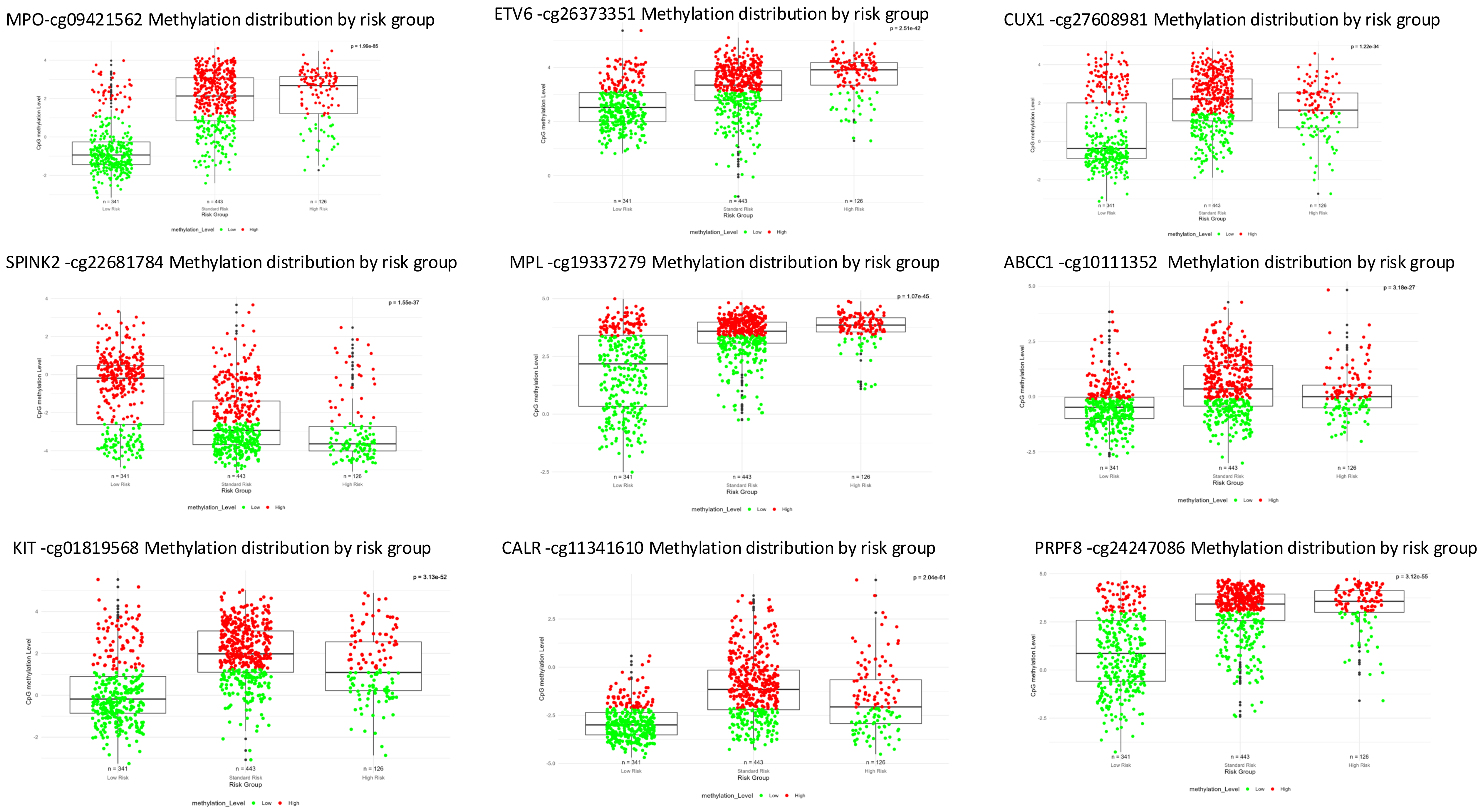

MPO-cg09421562 Methylation distribution by risk group
p = 1.99e-85
ETV6 -cg26373351 Methylation distribution by risk group
p = 2.51e-42
CUX1 -cg27608981 Methylation distribution by risk group
p = 1.22e-34
SPINK2 -cg22681784 Methylation distribution by risk group
p = 1.55e-37
MPL -cg19337279 Methylation distribution by risk group
p = 1.07e-45
ABCC1 -cg10111352 Methylation distribution by risk group
p = 3.18e-27
KIT -cg01819568 Methylation distribution by risk group
p = 3.13e-52
CALR -cg11341610 Methylation distribution by risk group
p = 2.04e-61
PRPF8 -cg24247086 Methylation distribution by risk group
p = 3.12e-55
CpG methylation Level
n = 341
Low Risk
n = 443
Standard Risk
n = 126
High Risk
Risk Group
methylation_Level
Low
High

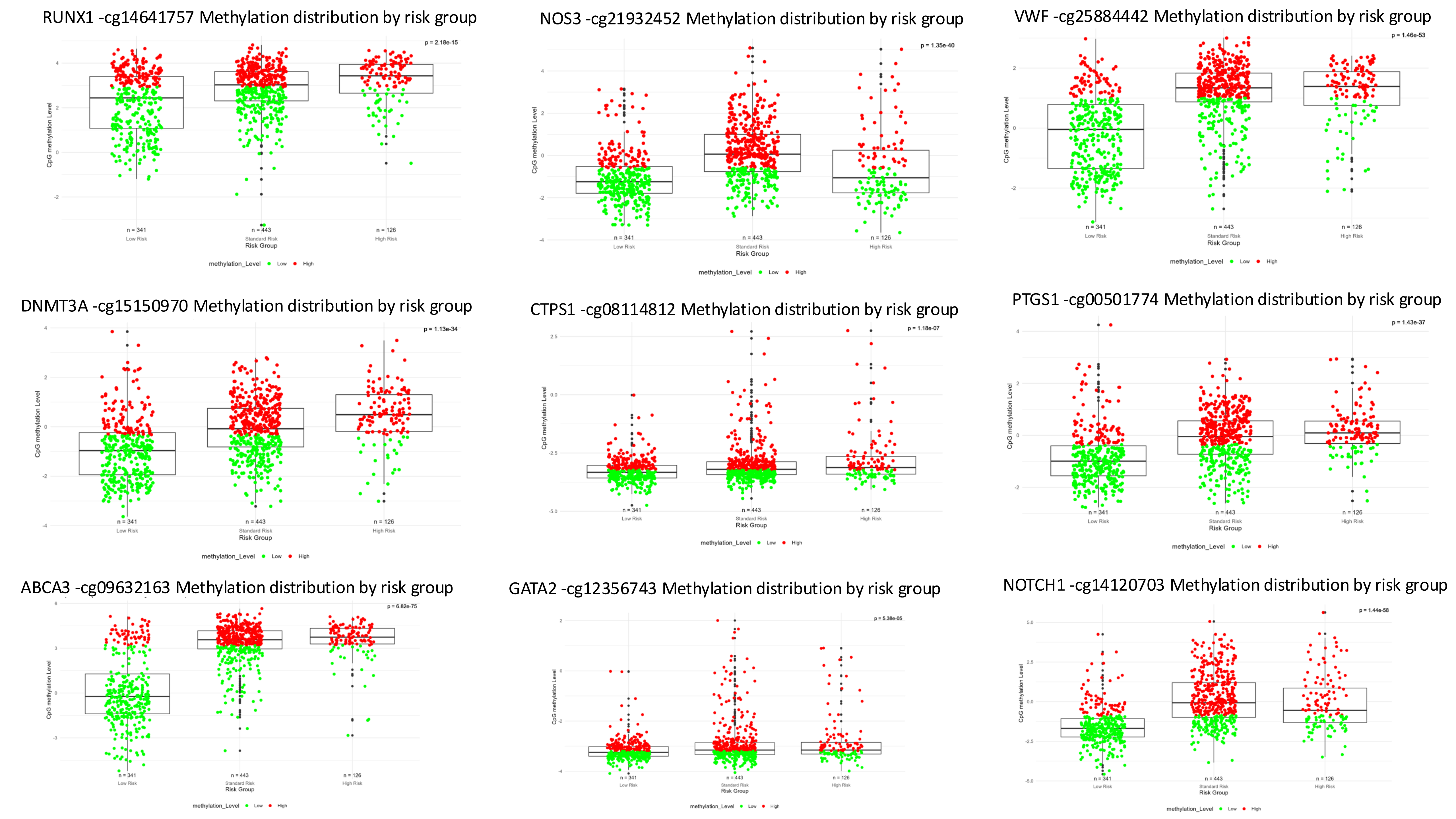

RUNX1 -cg14641757 Methylation distribution by risk group
p = 2.18e-15
NOS3 -cg21932452 Methylation distribution by risk group
p = 1.35e-40
VWF -cg25884442 Methylation distribution by risk group
p = 1.46e-53
DNMT3A -cg15150970 Methylation distribution by risk group
p = 1.13e-34
CTPS1 -cg08114812 Methylation distribution by risk group
p = 1.18e-07
PTGS1 -cg00501774 Methylation distribution by risk group
p = 1.43e-37
ABCA3 -cg09632163 Methylation distribution by risk group
p = 6.82e-75
GATA2 -cg12356743 Methylation distribution by risk group
p = 5.38e-05
NOTCH1 -cg14120703 Methylation distribution by risk group
p = 1.44e-58
CpG methylation Level
n = 341
Low Risk
n = 443
Standard Risk
n = 126
High Risk
Risk Group
methylation_Level Low High

DNMT3B -cg17475857 Methylation distribution by risk group

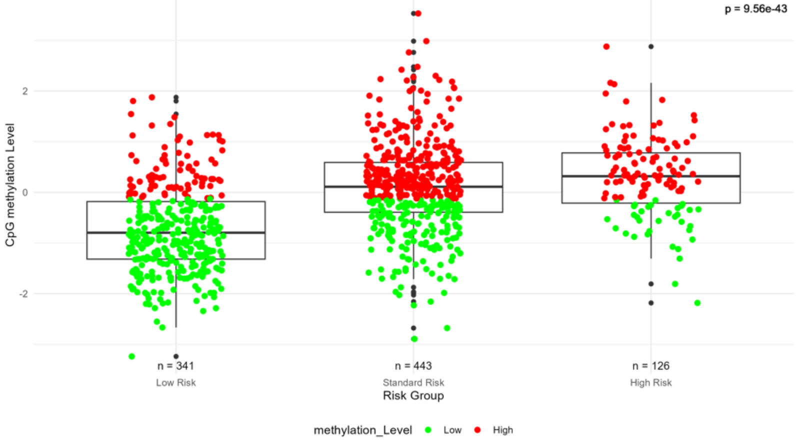

CREBBP -cg04141008 Methylation distribution by risk group

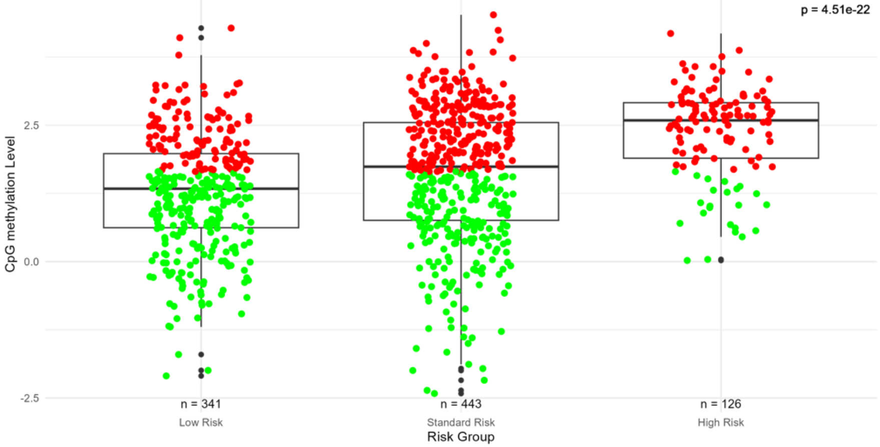

AKR1C1 -cg10158681 Methylation distribution by risk group

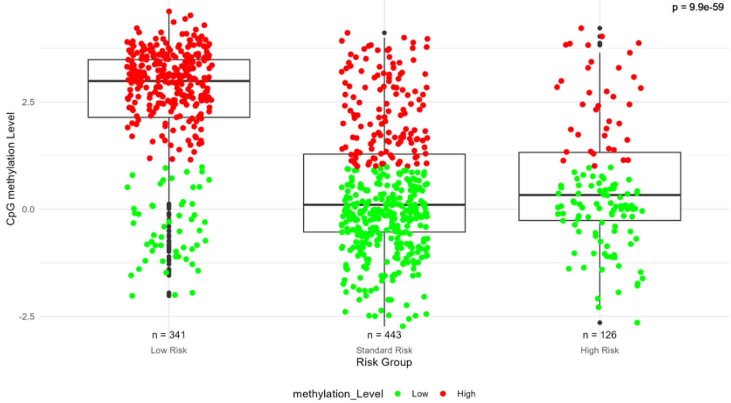

DPYSL3 -cg12807764 Methylation distribution by risk group

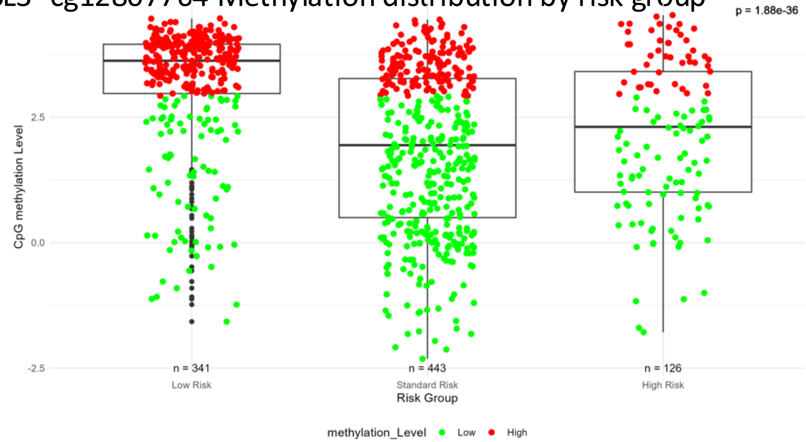

CD34 -cg03004102 Methylation distribution by risk group

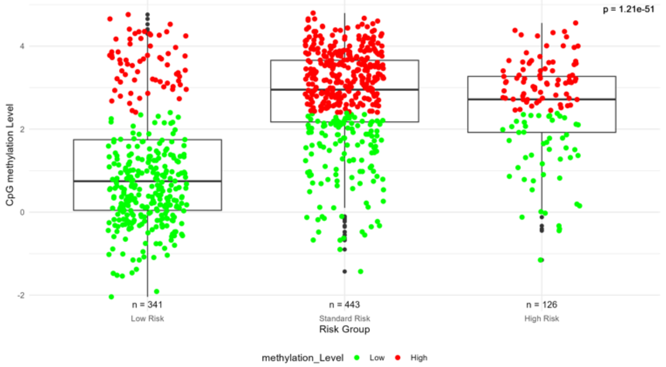

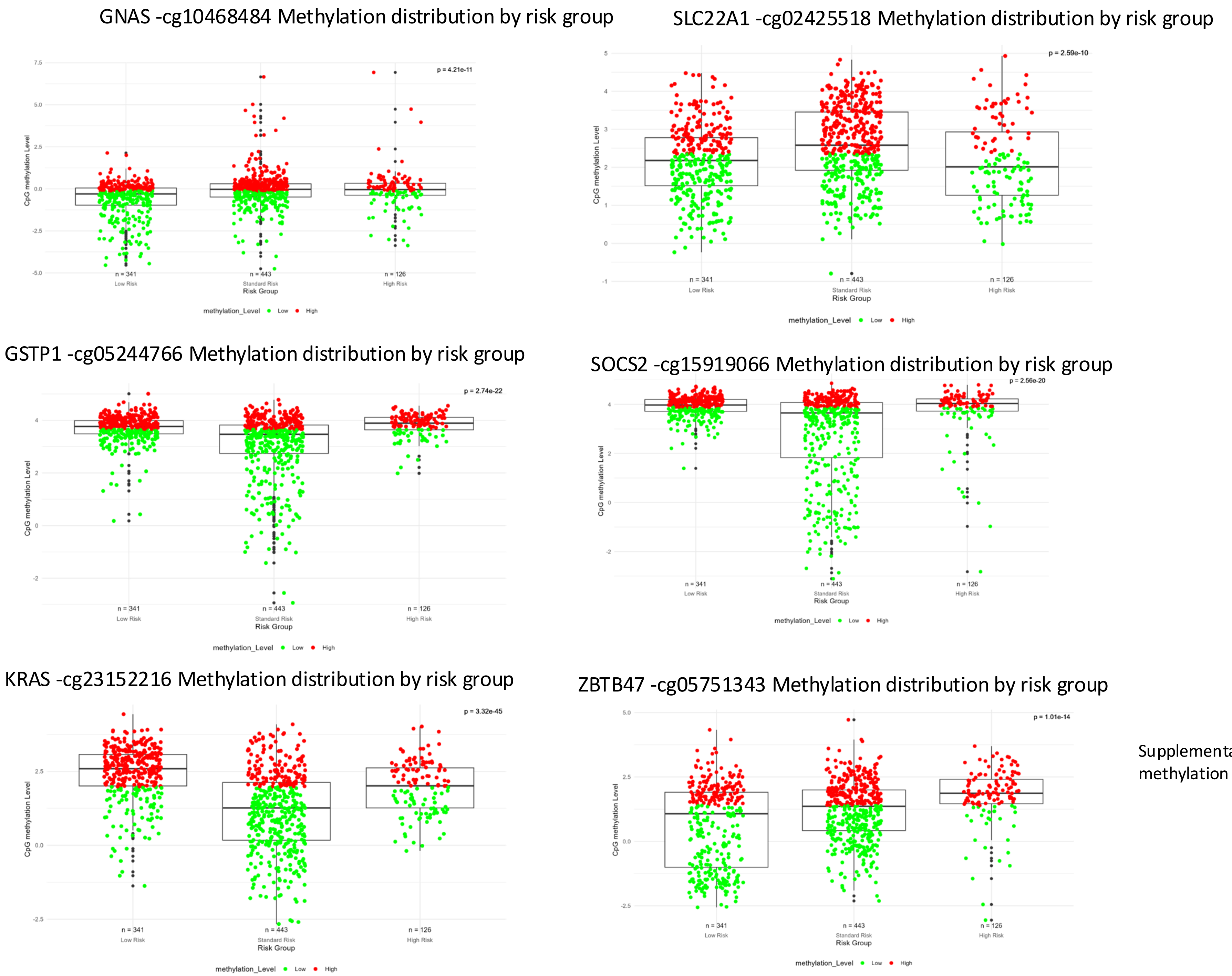

Supplementary Figure S2. Box plots showing association of DNA methylation levels of individual genes by risk groups.

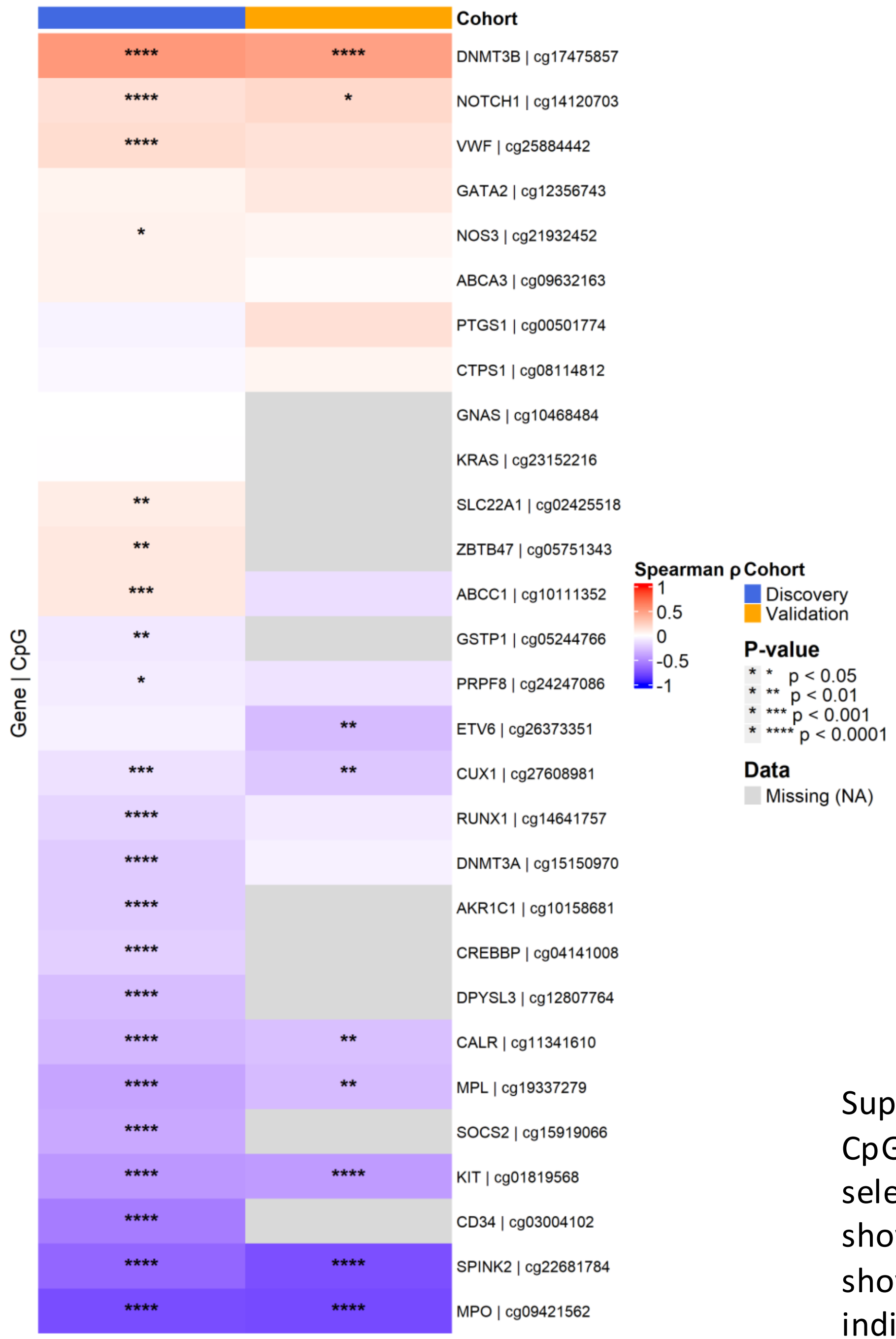

Supplementary Figure S3. Heatmap showing association gene expression with CpG methylation (discovery- RNAseq and validation cohort-U133A array) of selected CpGs that were associated with clinical outcome in Figure 1. Top bar shows the cohort (blue- discovery and orange validation cohort). p values are shown by * and direction of correlation blue negative and red positive. Grey indicates data not available in validation cohort.

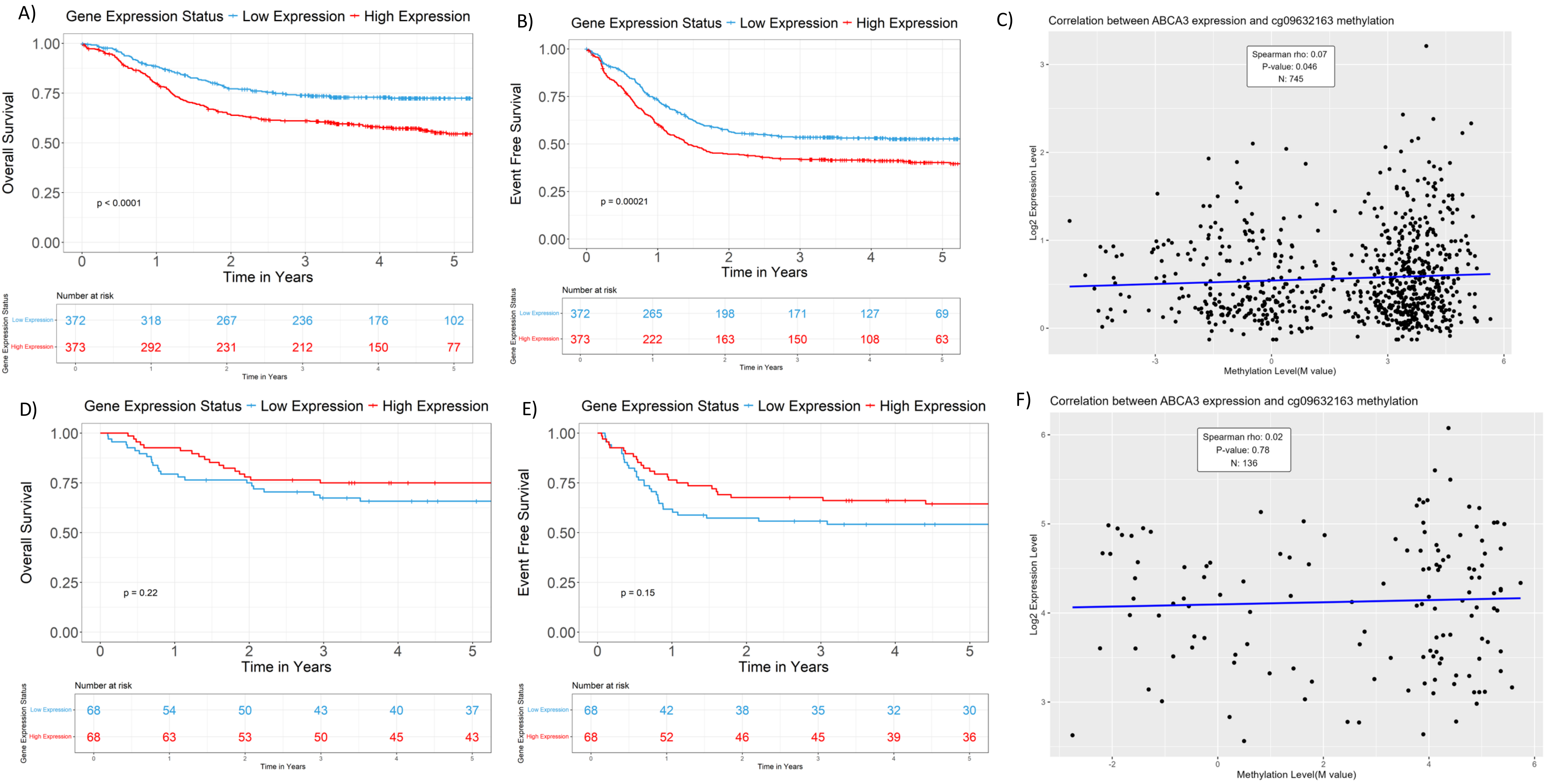

Supplementary Figure S4. Association of expression levels of ABCA3 with outcome in pediatric AML. Kaplan-Meier survival curves for by median expression values for OS and EFS in discovery cohort (A and B) and validation cohort (D and E). Correlation between ABCA3 Cg09632163 methylation levels and gene expression is shown in scatter plots for discovery (C)and validation cohorts (F)

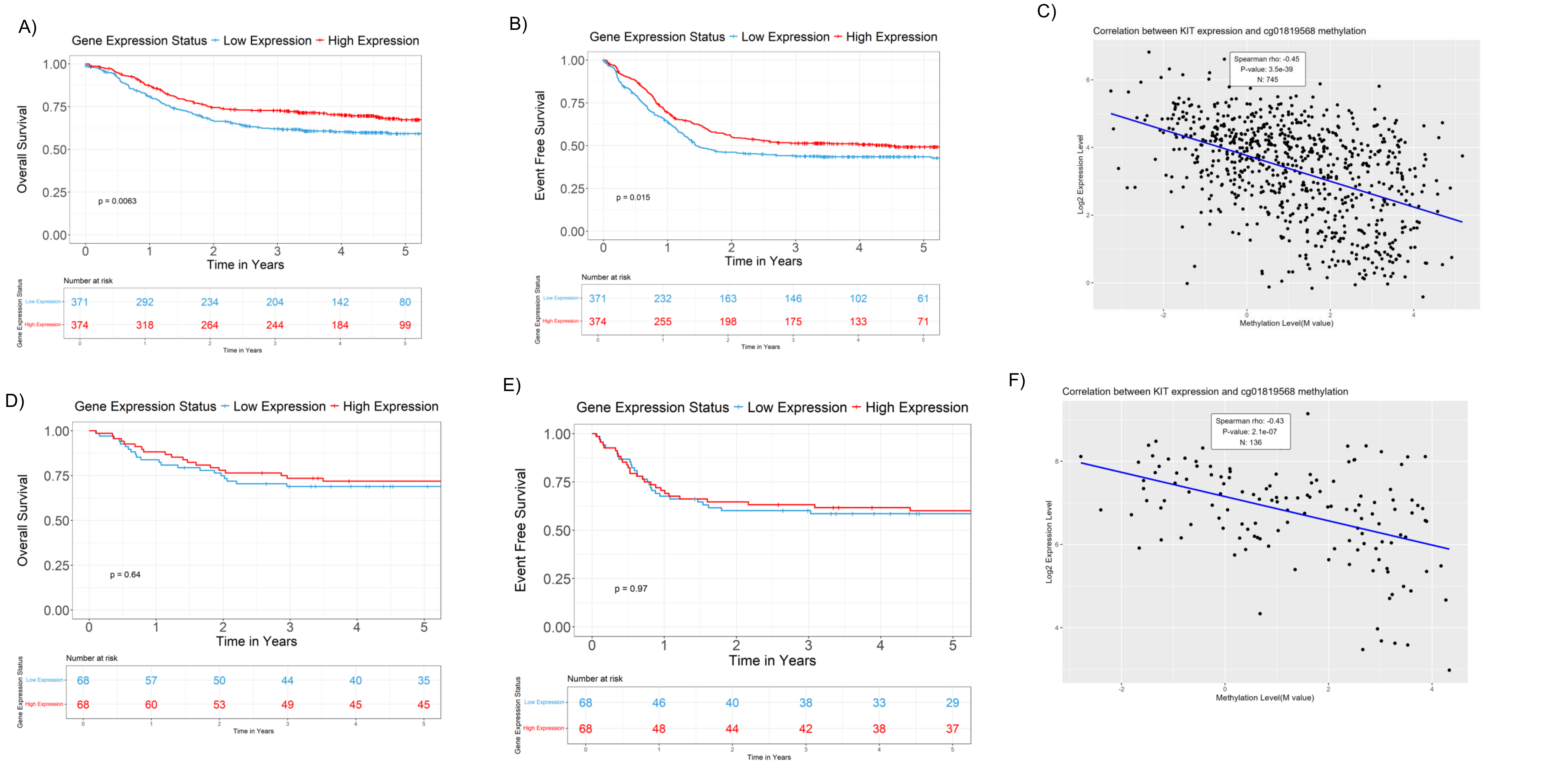

Supplementary Figure S5. Association of methylation levels of a KIT expression with outcome in pediatric AML. Kaplan-Meier survival curves for by median methylation values for OS and EFS in discovery cohort (A and B) and validation cohort (D and E). Correlation between MPO Cg09421562 methylation levels and gene expression is shown in scatter plots for discovery (C) and validation cohorts (F)

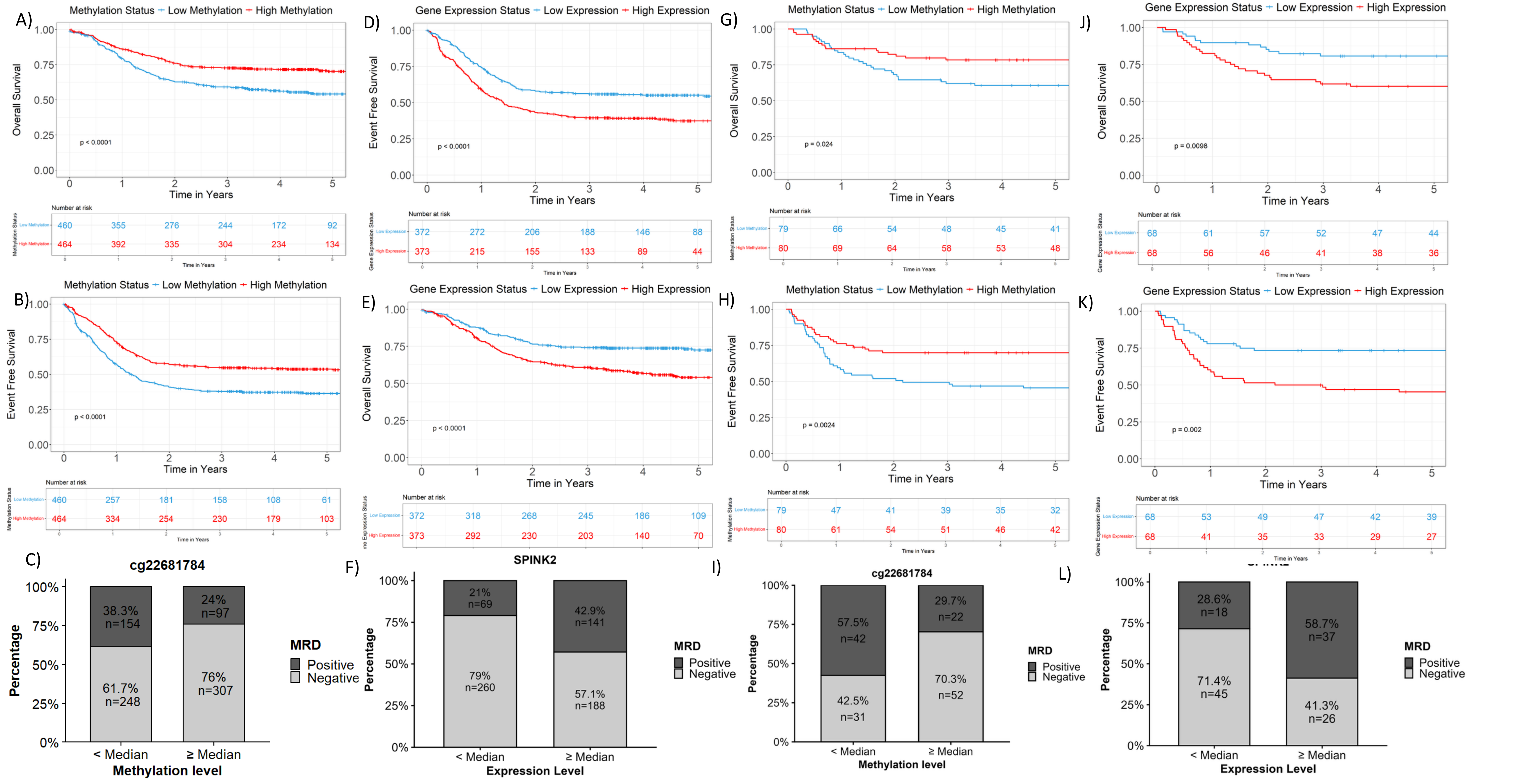

Supplementary Figure S6**.** Association of methylation levels (cg22681784) and Gene Expression SPINK2 with outcome in pediatric AML. Kaplan-Meier survival curves for by median methylation values (A-C) and SPIN Expression (D-F) for OS and EFS and MRD1 in discovery cohort and validation cohort (G-L). Stacked bar plot showing the proportion of who were MRD negative (light grey), or MRD positive (dark grey) by MPO methylation (by median) in discovery and validation cohorts (C and F in discovery and I and L in validation cohort).

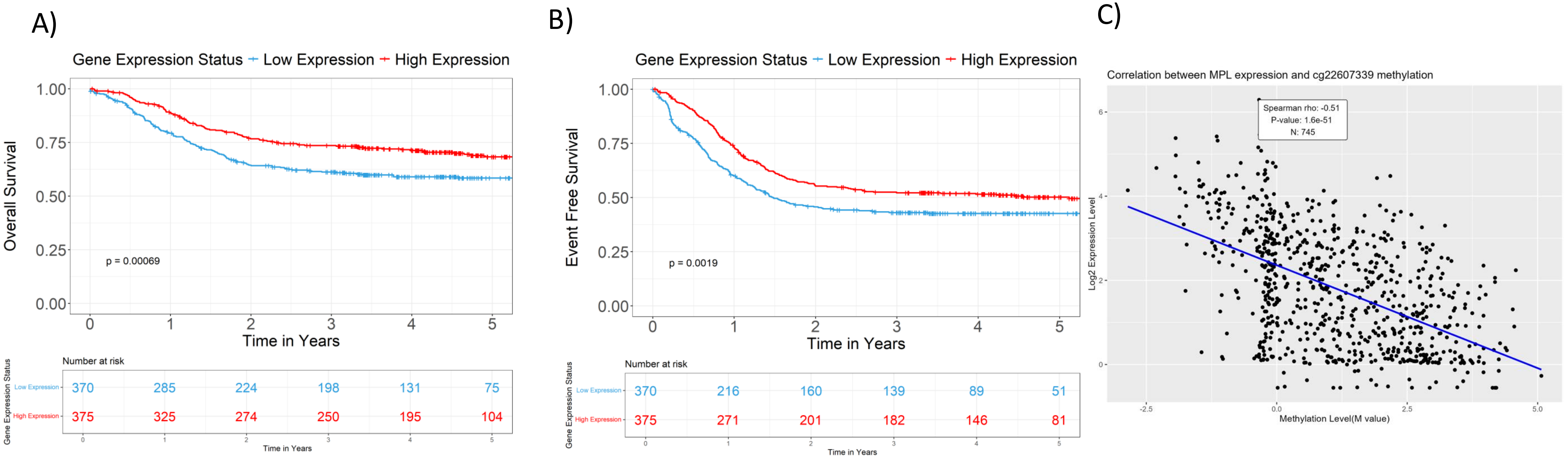

Supplementary Figure S7 . Association of MPL expression with low expression with OS (A) and EFS (B) in discovery cohort. C) Scatterplot showing correlation of MPL methylation with expression in discovery cohort.

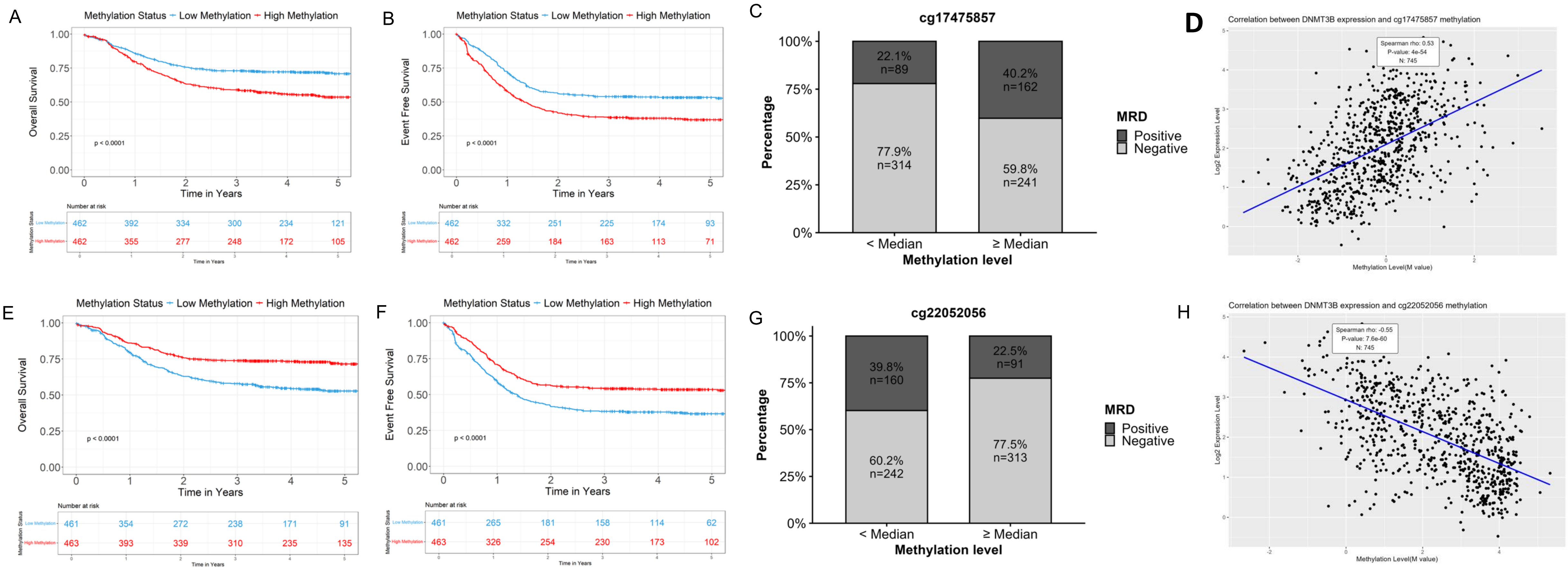

Supplementary Figure S8. Association of methylation levels of an intronic Cg17475857 and Cg22052056 in DNMT3B with outcome in pediatric AML. Kaplan-Meier survival curves for by median methylation values for OS and EFS in discovery cohort (A and B) for Cg17475857 and (E and F) for Cg22052056. Stacked bar plot showing the proportion of who were MRD negative (light grey), or MRD positive (dark grey) and Correlation between DNMT3B Cg17475857 and Cg22052056 methylation levels with gene expression is shown in scatter plots (D and H)

Supplementary Table S1: List of CpGs and genes investigated in this study

Gene Symbol	Gene name	Role	Gene type	Chromosome
ABCA3	ATP binding cassette subfamily A member 3	ADE metabolism	protein-coding	16
ABCB1	ATP binding cassette subfamily B member 1	ADE metabolism	protein-coding	7
ABCC1	ATP binding cassette subfamily C member 1	ADE metabolism	protein-coding	16
ABCC10	ATP binding cassette subfamily C member 10	ADE metabolism	protein-coding	6
ABCC11	ATP binding cassette subfamily C member 11	ADE metabolism	protein-coding	16
ABCC2	ATP binding cassette subfamily C member 2	ADE metabolism	protein-coding	10
ABCC3	ATP binding cassette subfamily C member 3	ADE metabolism	protein-coding	17
ABCC6	ATP binding cassette subfamily C member 6	ADE metabolism	protein-coding	16
ABCG2	[illegible] binding cassette subfamily G member 2 (Junior blood gro	ADE metabolism	protein-coding	4
ABL1	ABL proto-oncogene 1, non-receptor tyrosine kinase	AML Gene	protein-coding	9
ADGRG1	adhesion G protein-coupled receptor G1	Leukemic Stemness signature	protein-coding	16
AGER	advanced glycosylation end-product specific receptor	Leukemic Stemness signature	protein-coding	6
AIF1L	allograft inflammatory factor 1 like	Leukemic Stemness signature	protein-coding	9
AKR1A1	aldo-keto reductase family 1 member A1	ADE metabolism	protein-coding	1
AKR1B1	aldo-keto reductase family 1 member B	ADE metabolism	protein-coding	7
AKR1C1	aldo-keto reductase family 1 member C1	ADE metabolism	protein-coding	10
AKR1C2	aldo-keto reductase family 1 member C2	ADE metabolism	protein-coding	10
AKR1C3	aldo-keto reductase family 1 member C3	ADE metabolism/LSC	protein-coding	10
AKR1C4	aldo-keto reductase family 1 member C4	ADE metabolism	protein-coding	10
ANGPT1	angiopoietin 1	Leukemic Stemness signature	protein-coding	8
ARHGAP22	Rho GTPase activating protein 22	Leukemic Stemness signature	protein-coding	10
ASXL1	ASXL transcriptional regulator 1	AML Gene	protein-coding	20
ATP8B4	ATPase phospholipid transporting 8B4 (putative)	Leukemic Stemness signature	protein-coding	15
ATRX	ATRX chromatin remodeler	AML Gene	protein-coding	X
BCOR	BCL6 corepressor	AML Gene	protein-coding	X
BCORL1	BCL6 corepressor like 1	AML Gene	protein-coding	X
BEX3	brain expressed X-linked 3	Leukemic Stemness signature	protein-coding	X
BIVM	basic, immunoglobulin-like variable motif containing	Leukemic Stemness signature	protein-coding	13
BRAF	B-Raf proto-oncogene, serine/threonine kinase	AML Gene	protein-coding	7
CALR	calreticulin	AML Gene	protein-coding	19
CBL	Cbl proto-oncogene	AML Gene	protein-coding	11
CBLB	Cbl proto-oncogene B	AML Gene	protein-coding	3
CBLC	Cbl proto-oncogene C	AML Gene	protein-coding	19

CBR1	carbonyl reductase 1	ADE metabolism	protein-coding	21
CBR3	carbonyl reductase 3	ADE metabolism	protein-coding	21
CD34	CD34 molecule	Leukemic Stemness signature	protein-coding	1
CDA	cytidine deaminase	ADE metabolism	protein-coding	1
CDK6	cyclin dependent kinase 6	Leukemic Stemness signature	protein-coding	7
CDKN2A	cyclin dependent kinase inhibitor 2A	AML Gene	protein-coding	9
CEBPA	CCAAT enhancer binding protein alpha	AML Gene	protein-coding	19
CMPK1	cytidine/uridine monophosphate kinase 1	ADE metabolism	protein-coding	1
COL24A1	collagen type XXIV alpha 1 chain	Leukemic Stemness signature	protein-coding	1
CPXM1	carboxypeptidase X, M14 family member 1	Leukemic Stemness signature	protein-coding	20
CREBBP	CREB binding protein	AML Gene	protein-coding	16
CSF3R	colony stimulating factor 3 receptor	AML Gene	protein-coding	1
CTPS1	CTP synthase 1	ADE metabolism	protein-coding	1
CTPS2	CTP synthase 2	ADE metabolism	protein-coding	X
CUX1	cut like homeobox 1	AML Gene	protein-coding	7
CYBB/NOX2	cytochrome b-245 beta chain	ADE metabolism	protein-coding	X
CYP3A4	cytochrome P450 family 3 subfamily A member 4	ADE metabolism	protein-coding	7
CYP3A5	cytochrome P450 family 3 subfamily A member 5	ADE metabolism	protein-coding	7
DCK	deoxycytidine kinase	ADE metabolism	protein-coding	4
DCTD	dCMP deaminase	ADE metabolism	protein-coding	4
DIPK1B	divergent protein kinase domain 1B	Leukemic Stemness signature	protein-coding	9
DNMT3A	DNA methyltransferase 3 alpha	AML Gene	protein-coding	2
DNMT3B	DNA methyltransferase 3 beta	Leukemic Stemness signature	protein-coding	20
DPYSL3	dihydropyrimidinase like 3	Leukemic Stemness signature	protein-coding	5
EGFR	epidermal growth factor receptor	AML Gene	protein-coding	7
EMP1	epithelial membrane protein 1	Leukemic Stemness signature	protein-coding	12
EPDR1	ependymin related 1	Leukemic Stemness signature	protein-coding	7
ETV6	ETS variant transcription factor 6	AML Gene	protein-coding	12
EZH2	enhancer of zeste 2 polycomb repressive complex 2 subuni	AML Gene	protein-coding	7
FAM30A	family with sequence similarity 30 member A	Leukemic Stemness signature	ncRNA	14
FAS	Fas cell surface death receptor	ADE metabolism	protein-coding	10
FBXW7	F-box and WD repeat domain containing 7	AML Gene	protein-coding	4
FLT3	fms related receptor tyrosine kinase 3	AML Gene	protein-coding	13
FSCN1	fascin actin-bundling protein 1	Leukemic Stemness signature	protein-coding	7
GATA1	GATA binding protein 1	AML Gene	protein-coding	X

GATA2	GATA binding protein 2	Leukemic Stemness signature	protein-coding	3
GCSAML	germinal center associated signaling and motility like	Leukemic Stemness signature	protein-coding	1
GNAS	GNAS complex locus	AML Gene	protein-coding	20
GPSM1	G protein signaling modulator 1	Leukemic Stemness signature	protein-coding	9
GSTP1	glutathione S-transferase pi 1	ADE metabolism	protein-coding	11
GSTT1	glutathione S-transferase theta 1	ADE metabolism	protein-coding	22
GUCY1A1	guanylate cyclase 1 soluble subunit alpha 1	Leukemic Stemness signature	protein-coding	4
HOXA5	homeobox A5	Leukemic Stemness signature	protein-coding	7
HOXA6	homeobox A6	Leukemic Stemness signature	protein-coding	7
HOXA9	homeobox A9	Leukemic Stemness signature	protein-coding	7
HRAS	HRas proto-oncogene, GTPase	AML Gene	protein-coding	11
IDH1	isocitrate dehydrogenase (NADP(+)) 1	AML Gene	protein-coding	2
IDH2	isocitrate dehydrogenase (NADP(+)) 2	AML Gene	protein-coding	15
IKZF1	IKAROS family zinc finger 1	AML Gene	protein-coding	7
INKA1	inka box actin regulator 1	Leukemic Stemness signature	protein-coding	3
JAK2	Janus kinase 2	AML Gene	protein-coding	9
JAK3	Janus kinase 3	AML Gene	protein-coding	19
KCNK17	otassium two pore domain channel subfamily K member 1	Leukemic Stemness signature	protein-coding	6
KDM6A	lysine demethylase 6A	AML Gene	protein-coding	X
KIT	KIT proto-oncogene, receptor tyrosine kinase	AML Gene	protein-coding	4
KMT2A	lysine methyltransferase 2A	AML Gene	protein-coding	11
KMT2A	lysine methyltransferase 2A	AML Gene	protein-coding	11
KRAS	KRAS proto-oncogene, GTPase	AML Gene	protein-coding	12
LAPTM4B	lysosomal protein transmembrane 4 beta	Leukemic Stemness signature	protein-coding	8
MACROH2A2	macroH2A.2 histone	Leukemic Stemness signature	protein-coding	10
MAMDC2	MAM domain containing 2	Leukemic Stemness signature	protein-coding	9
MMRN1	multimerin 1	Leukemic Stemness signature	protein-coding	4
MPL	MPL proto-oncogene, thrombopoietin receptor	AML Gene	protein-coding	1
MPO	myeloperoxidase	ADE metabolism	protein-coding	17
MSH2	mutS homolog 2	ADE metabolism	protein-coding	2
MYC	MYC proto-oncogene, bHLH transcription factor	AML Gene	protein-coding	8
MYCN	MYCN proto-oncogene, bHLH transcription factor	Leukemic Stemness signature	protein-coding	2
MYD88	MYD88 innate immune signal transduction adaptor	AML Gene	protein-coding	3
NDUFS2	NADH:ubiquinone oxidoreductase core subunit S2	ADE metabolism	protein-coding	1
NDUFS3	NADH:ubiquinone oxidoreductase core subunit S3	ADE metabolism	protein-coding	11

NDUFS7	NADH:ubiquinone oxidoreductase core subunit S7	ADE metabolism	protein-coding	19
NF1	neurofibromin 1	AML Gene	protein-coding	17
NFKB1	nuclear factor kappa B subunit 1	ADE metabolism	protein-coding	4
NME1	NME/NM23 nucleoside diphosphate kinase 1	ADE metabolism	protein-coding	17
NME2	NME/NM23 nucleoside diphosphate kinase 2	ADE metabolism	protein-coding	17
NME3	NME/NM23 nucleoside diphosphate kinase 3	ADE metabolism	protein-coding	16
NOS1	nitric oxide synthase 1	ADE metabolism	protein-coding	12
NOS2	nitric oxide synthase 2	ADE metabolism	protein-coding	17
NOS3	nitric oxide synthase 3	ADE metabolism	protein-coding	7
NOTCH1	notch receptor 1	AML Gene	protein-coding	9
NPM1	nucleophosmin 1	AML Gene	protein-coding	5
NQO1	NAD(P)H quinone dehydrogenase 1	ADE metabolism	protein-coding	16
NR1I2	nuclear receptor subfamily 1 group I member 2	ADE metabolism	protein-coding	3
NRAS	NRAS proto-oncogene, GTPase	AML Gene	protein-coding	1
NT5C2	5'-nucleotidase, cytosolic II	ADE metabolism	protein-coding	10
NT5C3A	5'-nucleotidase, cytosolic IIIA	ADE metabolism	protein-coding	7
NTRK3	neurotrophic receptor tyrosine kinase 3	AML Gene	protein-coding	15
NYNRIN	NYN domain and retroviral integrase containing	Leukemic Stemness signature	protein-coding	14
PDGFRA	platelet derived growth factor receptor alpha	AML Gene	protein-coding	4
PHF6	PHD finger protein 6	AML Gene	protein-coding	X
POLA1	DNA polymerase alpha 1, catalytic subunit	ADE metabolism	protein-coding	X
POLA2	DNA polymerase alpha 2, accessory subunit	ADE metabolism	protein-coding	11
POLB	DNA polymerase beta	ADE metabolism	protein-coding	8
PRPF8	pre-mRNA processing factor 8	AML Gene	protein-coding	17
PRSS57	serine protease 57	Leukemic Stemness signature	protein-coding	19
PTEN	phosphatase and tensin homolog	AML Gene	protein-coding	10
PTGS1	prostaglandin-endoperoxide synthase 1	ADE metabolism	protein-coding	9
PTGS2	prostaglandin-endoperoxide synthase 2	ADE metabolism	protein-coding	1
PTPN11	protein tyrosine phosphatase non-receptor type 11	AML Gene	protein-coding	12
RAD21	RAD21 cohesin complex component	AML Gene	protein-coding	8
RALBP1	ralA binding protein 1	ADE metabolism	protein-coding	18
RB1	RB transcriptional corepressor 1	AML Gene	protein-coding	13
RRM1	ribonucleotide reductase catalytic subunit M1	ADE metabolism	protein-coding	11
RRM2	ribonucleotide reductase regulatory subunit M2	ADE metabolism	protein-coding	2
RUNX1	RUNX family transcription factor 1	AML Gene	protein-coding	21

SAMHD1	omain containing deoxynucleoside triphosphate triphosp	ADE metabolism	protein-coding	20
SETBP1	SET binding protein 1	AML Gene	protein-coding	18
SF3B1	splicing factor 3b subunit 1	AML Gene	protein-coding	2
SH2B3	SH2B adaptor protein 3	AML Gene	protein-coding	12
SHANK3	SH3 and multiple ankyrin repeat domains 3	Leukemic Stemness signature	protein-coding	22
SKIDA1	SKI/DACH domain containing 1	Leukemic Stemness signature	protein-coding	10
SLC22A1	solute carrier family 22 member 1	ADE metabolism	protein-coding	6
SLC22A5	solute carrier family 22 member 5	ADE metabolism	protein-coding	5
SLC28A1	solute carrier family 28 member 1	ADE metabolism	protein-coding	15
SLC28A3	solute carrier family 28 member 3	ADE metabolism	protein-coding	9
SLC29A1	solute carrier family 29 member 1 (Augustine blood group	ADE metabolism	protein-coding	6
SLCO1B1	olute carrier organic anion transporter family member 1B	ADE metabolism	protein-coding	12
SMC1A	structural maintenance of chromosomes 1A	AML Gene	protein-coding	X
SMC3	structural maintenance of chromosomes 3	AML Gene	protein-coding	10
SMIM24	small integral membrane protein 24	Leukemic Stemness signature	protein-coding	19
SOCS2	suppressor of cytokine signaling 2	Leukemic Stemness signature	protein-coding	12
SOD1	superoxide dismutase 1	ADE metabolism	protein-coding	21
SPINK2	serine peptidase inhibitor Kazal type 2	Leukemic Stemness signature	protein-coding	4
SPNS2	SPNS lysolipid transporter 2, sphingosine-1-phosphate	Leukemic Stemness signature	protein-coding	17
SRSF2	serine and arginine rich splicing factor 2	AML Gene	protein-coding	17
STAG2	stromal antigen 2	AML Gene	protein-coding	X
TET2	tet methylcytosine dioxygenase 2	AML Gene	protein-coding	4
TFPI	tissue factor pathway inhibitor	Leukemic Stemness signature	protein-coding	2
TNFRSF4	TNF receptor superfamily member 4	Leukemic Stemness signature	protein-coding	1
TOP2A	DNA topoisomerase II alpha	ADE metabolism	protein-coding	17
TOP2B	DNA topoisomerase II beta	ADE metabolism	protein-coding	3
TP53	tumor protein p53	AML Gene	protein-coding	17
U2AF1	U2 small nuclear RNA auxiliary factor 1	AML Gene	protein-coding	21
UGT1A1	UDP glucuronosyltransferase family 1 member A1	ADE metabolism	protein-coding	2
VWF	von Willebrand factor	Leukemic Stemness signature	protein-coding	12
WT1	WT1 transcription factor	AML Gene	protein-coding	11
XDH	xanthine dehydrogenase	ADE metabolism	protein-coding	2
ZBTB46	zinc finger and BTB domain containing 46	Leukemic Stemness signature	protein-coding	20
ZRSR2	finger CCCH-type, RNA binding motif and serine/arginine r	AML Gene	protein-coding	X

Supplementary Table S2. Association of Methylation of CpGs in candidate genes with clinical outcome (Unadjusted and Risk Adjusted Analysis) in discovery and validation cohorts

			Discovery (n= 924) Unadjusted OS analysis				Discovery (n= 924) Unadjusted EFS analysis				Discovery (n=924) Unadjusted MRD				Validation (n=159) Unadjusted OS analysis				Validation (n=159) Unadjusted EFS analysis				Validation (n=159) Unadjusted MRD				Discovery (n= 924) Risk adjusted OS analysis				Risk
Probe ID	CpG_ Region	Gene	HR	Lower 95%CI	Upper 95%CI	P-value 1	HR	Lower 95%CI	Upper 95%CI	P-value 1	OR	Lower 95%CI	Upper 95%CI	P-value 1	HR	Lower 95%CI	Upper 95%CI	P-value	HR	Lower 95%CI	Upper 95%CI	P-value	OR	Lower 95%CI	Upper 95%CI	P-value	HR	Lower 95%CI	Upper 95%CI	P-value	HR
cg01278797	Intron	ABCA3	1.43	1.25	1.64	1.45E-07	1.35	1.22	1.50	7.71E-09	1.29	1.10	1.54	0.00274	1.81	1.20	2.72	0.0046	1.61	1.17	2.22	0.0036	1.47	1.01	2.22	0.052	1.15	1.00	1.33	0.056	1.14
cg20915212	Intron	ABCA3	1.40	1.24	1.58	5.93E-08	1.30	1.18	1.43	1.75E-07	1.33	1.12	1.58	0.00136	1.83	1.23	2.73	0.0032	2.13	1.49	3.03	2.86E-05	1.88	1.19	3.05	0.008	1.12	0.97	1.29	0.124	1.08
cg04149930	Intron	ABCA3	1.31	1.20	1.44	3.57E-09	1.21	1.13	1.30	2.75E-07	1.38	1.21	1.57	1.04573E-06	1.49	1.19	1.88	0.0007	1.41	1.17	1.71	0.0004	1.41	1.09	1.86	0.012	1.05	0.94	1.17	0.364	1.00
cg09481056	Intron	ABCA3	1.25	1.17	1.34	2.00E-11	1.18	1.12	1.24	4.54E-10	1.22	1.12	1.33	1.29416E-05	1.43	1.14	1.80	0.0022	1.51	1.24	1.83	4.41E-05	1.35	1.08	1.70	0.009	1.02	0.93	1.12	0.671	0.99
cg09632163	Intron	ABCA3	1.23	1.16	1.30	1.30E-12	1.14	1.10	1.19	3.63E-10	1.13	1.06	1.22	0.00031	1.22	1.05	1.42	0.0089	1.25	1.10	1.42	0.00063	1.23	1.07	1.44	0.006	1.06	0.98	1.14	0.157	1.01
cg08449748	Intron	ABCA3	1.18	1.11	1.26	1.79E-07	1.12	1.07	1.18	8.24E-06	1.20	1.09	1.32	0.00013	1.35	1.11	1.65	0.0025	1.47	1.24	1.75	1.03E-05	1.77	1.36	2.37	4.723E-05	1.02	0.95	1.10	0.574	0.99
cg09263316	Intron	ABCA3	1.17	1.10	1.24	6.03E-07	1.08	1.03	1.14	0.0018	1.15	1.06	1.25	0.00108	1.31	1.12	1.54	0.0007	1.18	1.04	1.33	0.012	1.35	1.14	1.62	0.001	1.06	0.99	1.13	0.089	0.98
cg10111352	Intron	ABCC1	1.29	1.18	1.40	2.42E-09	1.20	1.12	1.29	1.70E-07	1.09	0.97	1.23	0.16	1.74	1.34	2.27	4.22E-05	1.54	1.24	1.92	0.0001	1.04	0.77	1.42	0.786	1.15	1.05	1.25	0.003	1.09
cg09109383	Intron	ABCC1	1.19	1.10	1.28	7.29E-06	1.13	1.06	1.20	8.98E-05	0.99	0.89	1.11	0.902	1.15	0.95	1.39	0.150	1.20	1.03	1.41	0.022	1.06	0.86	1.31	0.594	1.04	0.95	1.13	0.401	1.00
cg10158681	Promoter	AKR1C1	0.86	0.81	0.91	1.10E-06	0.90	0.86	0.94	2.25E-05	0.99	0.91	1.08	0.852	0.86	0.73	0.73	0.062	0.92	0.80	1.05	0.203	1.11	0.92	1.34	0.273	1.02	0.95	1.10	0.614	1.04
cg19867991	Exon	CALR	1.20	1.12	1.28	1.42E-07	1.16	1.09	1.22	4.12E-07	1.17	1.07	1.29	0.00112	1.45	1.19	1.78	0.00024	1.34	1.13	1.59	0.0007	1.27	1.00	1.63	0.053	1.03	0.95	1.11	0.524	1.02
cg04334723	3'UTR	CALR	1.20	1.11	1.28	7.01E-07	1.15	1.09	1.22	2.07E-06	1.42	1.28	1.58	4.90444E-11	1.61	1.33	1.95	1.17E-06	1.50	1.27	1.77	1.24E-06	1.86	1.42	2.49	1.307E-05	1.04	0.95	1.12	0.399	1.02
cg11341610	Promoter	CALR	1.17	1.11	1.24	1.61E-07	1.17	1.11	1.22	2.24E-09	1.16	1.06	1.26	0.0013	1.28	1.13	1.45	0.00013	1.25	1.12	1.39	6.27E-05	1.21	1.02	1.45	0.032	1.02	0.95	1.09	0.669	1.04
cg13466180	Intron	CALR	1.17	1.09	1.25	1.47E-05	1.15	1.09	1.22	6.25E-07	1.34	1.21	1.48	1.10766E-08	1.53	1.20	1.93	0.00046	1.44	1.20	1.73	0.0001	1.40	1.13	1.78	0.003	1.03	0.95	1.11	0.483	1.04
cg03004102	exon	CD34	1.22	1.13	1.32	1.63E-07	1.11	1.05	1.18	1.177	0.99	0.90	1.10	0.893	1.12	0.94	1.34	0.2187	1.10	0.94	1.28	0.222	0.88	0.71	1.08	0.208	1.01	0.92	1.10	0.858	0.94
cg04141008	Promoter	CREBBP	1.24	1.13	1.37	0.000012	1.13	1.05	1.23	1.226	1.42	1.24	1.64	4.44989E-07	1.29	1.00	1.68	0.0515	1.42	1.13	1.78	0.002	1.13	0.87	1.49	0.352	1.12	1.01	1.23	0.025	1.05
cg08114812	exon	CTPS1	1.26	1.13	1.39	1.75E-05	1.19	1.09	1.30	9.52E-05	1.36	1.14	1.62	0.001	1.36	1.10	1.10	0.0043	1.42	1.17	1.72	0.0004	1.64	1.09	2.77	0.035	1.11	0.99	1.24	0.069	1.08
cg27608981	Intron	CUX1	1.16	1.09	1.23	3.80E-06	1.08	1.03	1.13	1.133	1.09	1.01	1.19	0.038	1.27	1.07	1.51	0.0058	1.20	1.05	1.39	0.01	1.12	0.94	1.35	0.199	1.03	0.96	1.11	0.433	0.98
cg00856404	Promoter	DNMT3A	1.32	1.19	1.47	1.45E-07	1.20	1.10	1.31	5.06416E-05	1.04	0.88	1.22	0.67899	1.51	1.10	2.08	0.01192	1.53	1.16	2.01	0.002	1.45	0.97	2.21	0.076	1.10	0.96	1.25	0.160	1.01
cg20669908	Promoter	DNMT3A	1.32	1.20	1.45	3.21E-08	1.17	1.08	1.27	0.0002	1.05	0.89	1.22	0.57942	1.45	1.03	2.03	0.03155	1.50	1.13	2.00	0.005	1.51	0.99	2.35	0.059	1.12	1.00	1.26	0.058	1.00
cg09259843	3'UTR	DNMT3A	1.26	1.13	1.40	1.61E-05	1.17	1.08	1.28	0.0003	1.39	1.20	1.62	0.00001	2.03	1.48	2.78	1.31E-05	1.82	1.39	2.39	0.00002	1.80	1.27	2.63	0.001	0.96	0.85	1.09	0.571	0.94
cg15150970	Promoter	DNMT3A	1.25	1.15	1.36	1.18E-07	1.18	1.11	1.26	6.84E-07	1.22	1.09	1.38	0.0009	1.59	1.22	2.07	0.00057	1.45	1.16	1.80	0.0009	1.40	1.05	1.90	0.024	1.10	1.00	1.20	0.053	1.07
cg23009818	Promoter	DNMT3A	1.15	1.09	1.21	2.04E-07	1.09	1.04	1.14	0.0002	1.12	1.03	1.22	0.009	1.16	0.99	1.35	0.0664	1.22	1.07	1.39	0.004	1.59	1.21	2.21	0.002	1.10	1.04	1.17	0.001	1.06
cg08485187	Intron	DNMT3A	1.15	1.09	1.21	8.02E-07	1.10	1.05	1.15	0.0001	1.11	1.02	1.22	0.01932	1.15	0.95	1.39	0.1534	1.25	1.06	1.46	0.0062	1.59	1.16	2.29	0.007	1.09	1.03	1.16	0.003	1.06
cg17742416	Promoter	DNMT3A	1.14	1.08	1.21	1.28E-06	1.10	1.05	1.15	0.0001	1.18	1.09	1.29	0.00014	1.25	1.02	1.52	0.0288	1.30	1.09	1.54	0.003	1.79	1.25	2.69	0.003	1.08	1.02	1.15	0.008	1.05
cg17475857	Intron	DNMT3B	1.43	1.28	1.60	2.19E-10	1.30	1.19	1.43	1.84E-08	1.60	1.36	1.88	1.91015E-08	1.45	1.07	1.97	0.0171	1.59	1.21	2.09	0.0009	1.65	1.15	2.43	0.009	1.17	1.03	1.33	0.015	1.10
cg00300969	Intron	DNMT3B	1.34	1.20	1.49	1.51E-07	1.25	1.15	1.37	7.96E-07	1.73	1.47	2.05	8.63746E-11	1.51	1.13	2.01	0.0058	1.64	1.26	2.14	0.0002	1.88	1.30	2.82	0.001	1.12	0.99	1.26	0.078	1.08
cg24403338	Intron	DNMT3B	1.26	1.16	1.37	6.73E-08	1.16	1.08	1.24	0.00003	1.40	1.24	1.59	5.22623E-08	1.38	1.07	1.76	0.0116	1.37	1.11	1.69	0.003	1.38	1.06	1.83	0.019	1.06	0.96	1.17	0.239	1.00
cg22052056	Promoter	DNMT3B	0.83	0.78	0.89	2.04E-07	0.88	0.83	0.93	7.16E-06	0.77	0.70	0.85	4.78001E-07	0.78	0.66	0.92	0.0037	0.77	0.66	0.88	0.0003	0.61	0.48	0.77	6.578E-05	0.96	0.88	1.04	0.296	0.99
cg12807764	Intron	DPYSL3	0.85	0.80	0.91	0.00000177	0.90	0.86	0.95	0.0002	1.02	0.92	1.12	0.719	_	_	_	_	_	_	_	_	_	_	_	_	0.96	0.89	1.03	0.258	0.99
cg26373351	Intron	ETV6	1.52	1.34	1.73	4.32E-11	1.33	1.21	1.47	1.60E-08	1.46	1.24	1.74	1.04821E-05	1.94	1.32	2.86	0.0008	1.94	1.40	2.70	8.18E-05	1.30	0.87	1.98	0.211	1.25	1.10	1.43	0.001	1.13
cg05161923	Intron	ETV6	1.26	1.16	1.37	3.26E-08	1.13	1.07	1.20	0.00003	1.27	1.14	1.41	9.84726E-06	1.23	1.01	1.50	0.0384	1.25	1.06	1.48	0.007	1.26	1.04	1.55	0.022	1.13	1.03	1.23	0.006	1.03
cg12356743	Promoter	GATA2	1.29	1.17	1.44	1.51E-06	1.20	1.08	1.32	0.0004	1.25	1.04	1.51	0.02	1.33	1.06	1.67	0.0128	1.36	1.12	1.64	0.002	1.62	1.10	2.61	0.025	1.10	1.03	1.18	0.003	1.09
cg09024124	Promoter	GATA2	1.18	1.11	1.26	1.32E-07	1.12	1.06	1.18	0.0001	1.23	1.11	1.36	8.17231E-05	1.21	1.03	1.42	0.0235	1.31	1.14	1.50	8.50E-05	1.57	1.22	2.11	0.001	1.16	1.04	1.29	0.009	1.06
cg10468484	Exon	GNAS	1.23	1.12	1.34	0.000013	1.19	1.10	1.28	5.35E-06	1.13	0.99	1.29	0.065	1.29	0.92	1.82	0.1359	1.18	0.89	1.56	0.244	1.58	1.07	2.50	0.033	1.10	1.00	1.21	0.060	1.09
cg05244766	Promoter	GSTP1	0.81	0.74	0.89	0.00000771	0.88	0.81	0.95	0.0018	1.15	0.98	1.37	0.089	0.87	0.68	1.10	0.2405	0.85	0.69	1.05	0.125	1.39	1.00	2.05	0.066	0.89	0.81	0.98	0.020	0.96
cg24867302	Intron	KIT	1.24	1.14	1.34	1.63E-07	1.16	1.09	1.24	9.47E-06	1.05	0.93	1.17	0.432	1.22	0.99	1.49	0.0615	1.23	1.03	1.45	0.019	0.93	0.72	1.19	0.548	1.10	1.01	1.19	0.024	1.06
cg19041419	Intron	KIT	1.22	1.14	1.32	1.73E-07	1.11	1.05	1.18	0.0002	1.09	0.99	1.20	0.07	1.36	1.09	1.69	0.0065	1.30	1.09	1.54	0.003	1.11	0.91	1.37	0.312	1.02	0.93	1.11	0.734	0.95
cg01819568	Intron	KIT	1.22	1.14	1.30	2.92E-09	1.15	1.09	1.21	3.14E-07	1.05	0.96	1.15	0.274	1.31	1.09	1.56	0.0031	1.23	1.07	1.42	0.004	1.09	0.91	1.32	0.362	1.07	0.99	1.15	0.104	1.02
cg27154163	Promoter	KIT	1.21	1.11	1.33	1.83E-05	1.16	1.08	1.25	9.99E-05	1.25	1.08	1.45	0.002	1.25	0.94	1.67	0.1185	1.32	1.04	1.68	0.021	1.50	0.99	2.34	0.065	1.10	1.00	1.22	0.046	1.08
**cg06483432**	**Promoter**	**KIT**	**1.16**	**1.09**	**1.22**	**7.67E-07**	**1.13**	**1.08**	**1.19**	**9.67E-08**	**1.19**	**1.10**	**1.29**	**2.79504E-05**	**1.14**	**1.00**	**1.31**	**0.0587**	**1.13**	**1.00**	**1.27**	**0.048**	**1.21**	**1.02**	**1.44**	**0.029**	**1.08**	**1.02**	**1.15**	**0.014**	**1.08**
cg23152216	Promoter	KRAS	0.79	0.73	0.86	9.24E-09	0.85	0.80	0.91	2.22E-06	1.06	0.95	1.20	0.32	0.86	0.71	1.04	0.1301	0.92	0.78	1.08	0.308	1.23	0.98	1.55	0.085	0.91	0.83	1.00	0.044	0.97
cg19337279	Exon	MPL	1.26	1.15	1.38	3.85E-07	1.19	1.11	1.27	4.43E-07	1.24	1.11	1.39	0.0002	1.68	1.20	2.36	0.0028	1.46	1.14	1.86	0.002	1.42	1.11	1.88	0.009	1.03	0.92	1.14	0.622	1.02
cg22607339	Promoter	MPL	1.18	1.10	1.27	5.13E-06	1.14	1.07	1.21	1.20E-05	1.19	1.08	1.32	0.0006	1.39	1.15	1.68	0.0007	1.35	1.15	1.58	0.0003	1.59	1.25	2.06	0.0003	1.05	0.97	1.13	0.221	1.04
cg09421562	Intron	MPO	1.22	1.15	1.29	7.68E-11	1.15	1.10	1.20	8.37E-09	1.23	1.13	1.34	9.5346E-07	1.39	1.17	1.64	0.0001	1.32	1.16	1.51	4.55E-05	1.10	0.93	1.31	0.265	1.02	0.94	1.10	0.699	0.98
cg24499524	Intron	MPO	1.19	1.11	1.29	2.37E-06	1.13	1.07	1.21	0.00007	1.36	1.22	1.52	3.38943E-08	1.53	1.23	1.91	0.0001	1.46	1.22	1.76	0.00005	1.30	0.99	1.72	0.066	1.01	0.93	1.10	0.853	0.98
cg22331200	Exon	MPO	1.19	1.10	1.29	1.24E-05	1.12	1.05	1.20	0.0005	1.30	1.16	1.46	6.22875E-06	1.42	1.13	1.78	0.0025	1.42	1.16	1.74	0.0008	1.16	0.88	1.53	0.293	0.99	0.90	1.08	0.751	0.95
cg08058156	Intron	MPO	1.16	1.09	1.22	4.14E-07	1.10	1.05	1.15	0.00007	1.28	1.18	1.39	8.10057E-09	1.38	1.16	1.64	0.0003	1.33	1.15	1.53	0.00013	1.19	0.98	1.46	0.087	1.01	0.94	1.08	0.791	0.97
cg14619064	Exon	MPO	1.16	1.10	1.22	5.36E-09	1.10	1.06	1.15	1.20E-06	1.17	1.10	1.26	7.26371E-06	1.27	1.11	1.46	0.0006	1.26	1.12	1.41	0.00011	1.05	0.90	1.24	0.509	1.00	0.94	1.06	0.960	0.97
cg04266202	Intron	MPO	1.15	1.10	1.21	1.05E-09	1.11	1.07	1.15	2.22E-08	1.16	1.09	1.23	7.35858E-06	1.28	1.12	1.45	0.0002	1.26	1.14	1.39	1.23E-05	1.09	0.96	1.25	0.18	1.00	0.94	1.06	0.890	0.98
cg02668773	Intron	MPO	1.15	1.09	1.21	5.87E-07	1.09	1.05	1.14	0.00006	1.25	1.15	1.35	3.22116E-08	1.34	1.13	1.59	0.0009	1.31	1.13	1.51	0.0003	1.16	0.95	1.42	0.156	1.00	0.94	1.07	0.886	0.98
cg07110356	Exon	MPO	1.09	1.05	1.13	5.98E-07	1.06	1.03	1.09	0.00003	1.09	1.04	1.15	0.00021	1.14	1.05	1.25	0.0034	1.14	1.06	1.23	0.0007	1.03	0.93	1.14	0.562	0.99	0.95	1.03	0.642	0.98
cg21932452	Intron	NOS3	1.21	1.13	1.30	6.33E-08	1.12	1.06	1.19	0.0001	1.01	0.91	1.12	0.862	1.28	1.04	1.58	0.0208	1.21	1.02	1.44	0.032	1.02	0.80	1.30	0.88	1.09	1.01	1.17	0.035	1.02
cg14120703	Intron	NOTCH1	1.22	1.15	1.29	2.17E-11	1.16	1.10	1.22	2.41E-09	1.26	1.15	1.37	3.68882E-07	1.39	1.17	1.64	0.0001	1.40	1.22	1.62	3.04E-06	1.45	1.16	1.86	0.002	1.09	1.02	1.16	0.015	1.05
cg14410964	Intron	NOTCH1	1.19	1.11	1.28	2.50E-06	1.09	1.03	1.16	0.004	1.14	1.03	1.27	0.014	1.34	1.06	1.70	0.0149	1.51	1.24	1.84	4.64E-05	1.10	0.83	1.48	0.507	1.07	0.98	1.16	0.127	0.99
cg14217861	Intron	NOTCH1	1.16	1.09	1.23	1.55E-06	1.12	1.07	1.18	3.14E-06	1.10	1.02	1.19	0.02	1.15	1.00	1.32	0.057	1.19	1.05	1.35	0.006	1.18	1.01	1.40	0.042	1.03	0.97	1.10	0.299	1.03
cg24247086	Promoter	PRPF8	1.16	1.09	1.23	7.59E-06	1.06	1.01	1.11	0.022	1.29	1.18	1.41	7.12514E-08	1.40	1.17	1.68	0.0002	1.39	1.20	1.61	1.14E-05	1.47	1.23	1.80	5.278E-05	0.96	0.90	1.04	0.349	0.89
cg19805377	Promoter	PRPF8	1.13	1.07	1.19	1.76E-05	1.05	1.00	1.10	0.038	1.14	1.06	1.23	0.0007	1.28	1.12	1.47	0.0003	1.26	1.12	1.41	7.08E-05	1.29	1.11	1.51	0.002	1.00	0.94	1.06	0.911	0.93
cg00501774	Promoter	PTGS1	1.24	1.12	1.36	1.69E-05	1.14	1.05	1.23	0.0013	1.43	1.24	1.66	7.74995E-07	1.51	1.12	2.04	0.0066	1.47	1.14	1.90	0.003	2.17	1.48	3.29	0.0001	1.03	0.92	1.16	0.589	0.98
cg14641757	Intron	RUNX1	1.29	1.17	1.42	3.79E-07	1.15	1.07	1.24	0.0002	1.32	1.16	1.51	3.36114E-05	1.64	1.18	2.27	0.0029	1.34	1.06	1.69	0.013	1.31	1.00	1.76	0.057	1.16	1.04	1.29	0.006	1.06
cg02425518	exon	SLC22A1	1.28	1.15	1.42	9.62E-06	1.17	1.07	1.28	0.0004	0.99	0.86	1.15	0.907	1.27	0.91	0.91	0.1626	1.35	1.02	1.78	0.037	0.91	0.64	1.30	0.601	1.18	1.06	1.31	0.003	1.10
cg15919066	exon	SOCS2	0.87	0.82	0.92	0.0000061	0.90	0.85	0.95	0.0001	1.22	1.09	1.39	0.001	0.91	0.77	1.07	0.2666	0.91	0.79	1.04	0.158	1.34	1.05	1.78	0.027	0.95	0.89	1.02	0.164	0.98
cg22681784	Intron	SPINK2	0.87	0.82	0.92	0.00000305	0.88	0.84	0.92	1.19E-07	0.85	0.78	0.92	7.33762E-05	0.81	0.68	0.96	0.015	0.78	0.67	0.90	0.001	0.74	0.61	0.88	0.001	0.98	0.91	1.04	0.442	0.96
cg25884442	Intron	VWF	1.30	1.18	1.43	7.84E-08	1.18	1.09	1.27	1.22E-05	1.30	1.15	1.48	5.50273E-05	2.13	1.40	3.24	0.000	1.70	1.25	2.31	0.0007	1.45	1.04	2.05	0.032	1.03	0.93	1.15	0.552	0.98
cg05751343	Intron	ZBTB47	1.22	1.12	1.32	0.0000057	1.17	1.10	1.25	1.94E-06	1.24	1.11	1.40	0.0002	1.52	1.16	2.00	0.003	1.30	1.06	1.60	0.012	1.26	0.98	1.65	0.079	1.13	1.03	1.24	0.011	1.11

Discovery (n= 924) adjusted EFS analysis			Discovery (n= 924) Risk adjusted MRD				Validation (n=159) Risk adjusted OS analysis				Validation (n=159) Risk adjusted EFS analysis				Validation (n=159) Risk adjusted MRD				Discovery Validation DNA methylation and Gene expression correlation (un adjusted)			
Lower 95%CI	Upper 95%CI	P-value	OR	Lower 95%CI	Upper 95%CI	P-value	HR	Lower 95%CI	Upper 95%CI	P-value	HR	Lower 95%CI	Upper 95%CI	P-value	OR	Lower 95%CI	Upper 95%CI	P-value	ρ	P-value	ρ	P-value
1.02	1.27	0.021	1.02	0.85	1.24	0.814	1.41	0.93	2.16	0.109	1.31	0.94	1.83	0.108	0.95	0.61	1.50	0.84	0.100	0.013	0.010	0.912
0.97	1.22	0.171	0.99	0.81	1.21	0.952	1.33	0.84	2.10	0.221	1.82	1.21	2.76	0.004	1.06	0.60	1.86	0.85	0.080	0.023	0.030	0.772
0.92	1.10	0.965	1.17	1.00	1.37	0.044	1.28	0.97	1.69	0.077	1.23	0.98	1.55	0.072	0.96	0.69	1.34	0.83	0.140	0.000	0.050	0.525
0.92	1.06	0.817	0.99	0.87	1.12	0.842	1.17	0.89	1.54	0.269	1.35	1.07	1.69	0.010	0.89	0.64	1.22	0.50	0.100	0.009	0.060	0.453
0.95	1.06	0.834	0.94	0.85	1.03	0.209	1.03	0.85	1.24	0.773	1.12	0.96	1.30	0.161	0.93	0.74	1.15	0.52	0.070	0.050	0.020	0.781
0.94	1.06	0.861	1.05	0.94	1.17	0.372	1.12	0.89	1.40	0.341	1.28	1.06	1.56	0.013	1.32	0.97	1.83	0.08	0.040	0.278	0.140	0.110
0.93	1.04	0.568	1.07	0.98	1.18	0.143	1.15	0.98	1.35	0.080	1.18	1.04	1.33	0.012	1.15	0.95	1.41	0.16	0.180	0.000	0.050	0.532
1.01	1.17	0.028	0.97	0.84	1.11	0.623	2.05	1.51	2.78	4.16E-06	1.68	1.32	2.14	0.00003	1.08	0.76	1.55	0.66	0.120	0.001	-0.140	0.099
0.94	1.08	0.930	0.90	0.79	1.02	0.091	1.13	0.91	1.40	0.258	1.22	1.02	1.46	0.031	1.03	0.79	1.33	0.85	-0.294	0.000	-0.300	0.000
0.98	1.11	0.183	1.24	1.11	1.39	0.0002	0.95	0.79	1.13	0.544	1.03	0.88	1.19	0.745	1.56	1.21	2.06	0.00	-0.221	0.000	_	_
0.95	1.08	0.633	1.04	0.93	1.17	0.483	1.33	1.08	1.63	0.007	1.21	1.01	1.44	0.037	1.08	0.82	1.42	0.59	-0.340	0.000	-0.290	0.001
0.96	1.09	0.529	1.24	1.11	1.40	0.0003	1.42	1.14	1.78	0.002	1.32	1.09	1.60	0.004	1.41	1.06	1.91	0.02	-0.160	0.000	-0.160	0.056
0.98	1.10	0.207	1.03	0.93	1.15	0.550	1.24	1.08	1.42	0.0025	1.19	1.06	1.33	0.004	1.10	0.90	1.34	0.35	-0.310	0.000	-0.270	0.001
0.98	1.11	0.159	1.17	1.05	1.31	0.004	1.30	1.03	1.65	0.0258	1.27	1.05	1.53	0.012	1.15	0.91	1.47	0.25	-0.150	0.000	-0.040	0.674
0.87	1.00	0.064	0.76	0.67	0.87	0.0001	1.06	0.87	1.30	0.536	1.03	0.87	1.22	0.725	0.70	0.52	0.90	0.01	-0.566	0.000	_	_
0.97	1.14	0.229	1.23	1.07	1.43	0.0039	1.14	0.89	1.46	0.293	1.30	1.04	1.61	0.020	0.99	0.73	1.34	0.94	-0.205	0.000	_	_
0.98	1.19	0.111	1.16	0.96	1.39	0.119	1.34	1.06	1.71	0.016	1.38	1.11	1.71	0.003	1.42	0.96	2.31	0.10	-0.034	0.359	0.053	0.539
0.92	1.03	0.408	0.99	0.89	1.09	0.818	1.15	0.94	1.42	0.180	1.07	0.90	1.27	0.432	0.85	0.65	1.08	0.19	-0.128	0.000	-0.240	0.005
0.91	1.12	0.841	0.81	0.66	0.99	0.043	1.58	1.05	2.37	0.028	1.55	1.11	2.18	0.010	1.33	0.81	2.22	0.26	-0.150	0.000	-0.010	0.930
0.91	1.11	0.962	0.84	0.70	1.01	0.066	1.43	0.93	2.19	0.101	1.49	1.04	2.12	0.028	1.39	0.82	2.36	0.22	-0.150	0.000	-0.040	0.679
0.85	1.04	0.246	1.12	0.94	1.34	0.196	1.68	1.15	2.44	0.007	1.48	1.07	2.04	0.017	1.05	0.66	1.65	0.83	-0.030	0.418	0.140	0.093
0.99	1.15	0.089	1.02	0.89	1.16	0.770	1.36	1.03	1.79	0.030	1.25	1.00	1.58	0.055	1.05	0.73	1.49	0.80	-0.220	0.000	-0.060	0.470
1.01	1.11	0.029	1.04	0.95	1.14	0.378	1.01	0.84	1.20	0.953	1.08	0.92	1.26	0.352	1.20	0.90	1.66	0.22	-0.049	0.179	0.096	0.266
1.00	1.12	0.033	1.03	0.93	1.13	0.557	1.01	0.82	1.23	0.957	1.11	0.93	1.32	0.246	1.29	0.93	1.86	0.14	-0.050	0.165	0.160	0.066
1.00	1.10	0.075	1.09	0.99	1.19	0.085	1.05	0.84	1.32	0.653	1.11	0.91	1.36	0.305	1.22	0.84	1.84	0.30	-0.080	0.038	0.040	0.608
0.99	1.22	0.066	1.30	1.08	1.56	0.0049	1.27	0.89	1.80	0.186	1.36	0.99	1.86	0.059	1.19	0.77	1.86	0.43	0.530	0.000	0.490	0.000
0.98	1.20	0.120	1.43	1.20	1.72	0.0001	1.20	0.84	1.72	0.326	1.41	1.04	1.92	0.029	1.39	0.90	2.18	0.14	0.570	0.000	0.550	0.000
0.92	1.08	0.956	1.18	1.03	1.36	0.0192	1.15	0.87	1.52	0.314	1.17	0.93	1.48	0.185	1.03	0.74	1.43	0.88	0.570	0.000	0.490	0.000
0.93	1.06	0.801	0.91	0.81	1.02	0.107	0.95	0.77	1.16	0.604	0.90	0.75	1.07	0.237	0.85	0.65	1.12	0.25	-0.550	0.000	-0.600	0.000
0.94	1.06	0.868	1.16	1.04	1.30	0.008	_	_	_	_	_	_	_	_	_	_	_	_	-0.283	0.000	_	_
1.02	1.26	0.020	1.15	0.96	1.38	0.143	1.56	1.04	2.34	0.033	1.62	1.14	2.30	0.007	0.73	0.43	1.20	0.23	-0.060	0.097	-0.290	0.001
0.97	1.10	0.281	1.12	1.00	1.26	0.053	0.97	0.78	1.20	0.778	1.04	0.86	1.25	0.692	0.86	0.65	1.12	0.28	-0.020	0.506	-0.240	0.005
0.98	1.21	0.116	1.08	0.88	1.31	0.459	1.20	0.94	1.54	0.150	1.25	1.01	1.53	0.039	1.38	0.93	2.18	0.13	0.060	0.123	0.120	0.181
1.00	1.12	0.042	1.13	1.02	1.26	0.024	1.05	0.88	1.27	0.569	1.18	1.02	1.37	0.030	1.31	1.01	1.76	0.05	0.040	0.297	0.090	0.310
1.00	1.18	0.037	1.01	0.88	1.16	0.879	1.16	0.80	1.69	0.425	1.07	0.79	1.45	0.652	1.30	0.85	2.12	0.25	-0.002	0.966	_	_
0.88	1.05	0.382	1.22	1.03	1.47	0.027	0.77	0.60	0.99	0.045	0.77	0.62	0.96	0.021	1.25	0.88	1.88	0.24	-0.097	0.008	_	_
0.99	1.13	0.103	0.94	0.83	1.06	0.306	1.17	0.94	1.46	0.151	1.19	1.00	1.42	0.057	0.85	0.62	1.13	0.26	-0.334	0.000	-0.370	0.000
0.89	1.01	0.109	0.91	0.81	1.02	0.095	1.25	0.98	1.60	0.077	1.20	0.99	1.45	0.062	0.87	0.66	1.14	0.32	-0.440	0.000	-0.490	0.000
0.96	1.09	0.494	0.90	0.81	1.00	0.061	1.27	1.04	1.55	0.018	1.18	1.01	1.38	0.043	0.96	0.75	1.21	0.71	-0.450	0.000	-0.430	0.000
0.99	1.17	0.078	1.10	0.94	1.28	0.222	1.04	0.77	1.40	0.789	1.13	0.88	1.46	0.330	1.11	0.70	1.79	0.66	-0.155	0.000	-0.060	0.486
**1.03**	**1.14**	**0.003**	**1.08**	**0.99**	**1.18**	**0.080**	1.03	0.90	1.19	0.659	**1.03**	**0.91**	**1.17**	**0.656**	**1.03**	**0.84**	**1.25**	**0.80**	**-0.080**	**0.029**	**0.119**	**0.167**
0.90	1.05	0.509	1.26	1.10	1.45	0.001	0.86	0.70	1.07	0.177	0.96	0.80	1.15	0.632	1.43	1.09	1.93	0.01	-0.003	0.928	_	_
0.94	1.10	0.668	1.00	0.88	1.15	0.995	1.38	0.96	1.98	0.085	1.24	0.95	1.62	0.108	0.98	0.72	1.36	0.89	-0.390	0.000	-0.290	0.001
0.97	1.10	0.248	1.03	0.92	1.16	0.559	1.22	0.99	1.49	0.058	1.20	1.01	1.43	0.035	1.36	1.02	1.86	0.04	-0.510	0.000	-0.500	0.000
0.92	1.04	0.516	1.03	0.92	1.15	0.605	1.29	1.07	1.55	0.009	1.22	1.05	1.42	0.009	0.81	0.63	1.01	0.07	-0.770	0.000	-0.780	0.000
0.91	1.05	0.521	1.15	1.02	1.31	0.022	1.29	1.01	1.64	0.040	1.23	0.99	1.52	0.057	0.86	0.61	1.20	0.39	-0.580	0.000	-0.570	0.000
0.88	1.02	0.179	1.11	0.97	1.27	0.119	1.21	0.95	1.55	0.120	1.19	0.96	1.48	0.118	0.81	0.57	1.12	0.21	-0.580	0.000	-0.510	0.000
0.92	1.03	0.322	1.12	1.01	1.23	0.025	1.21	1.00	1.46	0.047	1.17	0.99	1.37	0.066	0.89	0.69	1.14	0.37	-0.600	0.000	-0.580	0.000
0.92	1.02	0.224	1.03	0.94	1.12	0.561	1.20	1.03	1.39	0.021	1.17	1.03	1.33	0.017	0.83	0.67	1.02	0.08	-0.770	0.000	-0.720	0.000
0.94	1.03	0.436	1.00	0.91	1.09	0.947	1.22	1.06	1.41	0.007	1.20	1.07	1.34	0.002	0.90	0.75	1.06	0.22	-0.760	0.000	-0.760	0.000
0.93	1.03	0.341	1.10	1.01	1.21	0.034	1.18	0.98	1.42	0.082	1.15	0.98	1.35	0.096	0.88	0.68	1.12	0.30	-0.620	0.000	-0.590	0.000
0.95	1.01	0.145	1.00	0.95	1.06	0.948	1.08	0.98	1.19	0.108	1.08	0.99	1.17	0.086	0.89	0.78	1.02	0.10	-0.720	0.000	-0.640	0.000
0.95	1.08	0.627	0.91	0.81	1.02	0.105	1.21	0.99	1.48	0.069	1.14	0.96	1.36	0.132	0.89	0.67	1.18	0.42	0.070	0.041	0.050	0.596
0.99	1.11	0.125	1.14	1.03	1.27	0.010	1.24	1.04	1.49	0.019	1.27	1.09	1.49	0.003	1.21	0.93	1.60	0.15	0.160	0.000	0.200	0.019
0.93	1.06	0.831	1.00	0.89	1.12	0.987	1.14	0.87	1.51	0.337	1.39	1.11	1.74	0.005	0.76	0.52	1.10	0.16	0.040	0.248	0.200	0.017
0.98	1.09	0.251	0.97	0.89	1.06	0.565	1.02	0.88	1.18	0.770	1.08	0.95	1.23	0.244	1.03	0.85	1.24	0.77	0.019	0.603	0.198	0.021
0.84	0.95	0.000	1.14	1.02	1.27	0.019	1.23	1.01	1.50	0.044	1.26	1.07	1.49	0.007	1.19	0.96	1.50	0.11	-0.080	0.027	-0.120	0.164
0.89	0.98	0.010	1.02	0.93	1.12	0.660	1.15	1.00	1.32	0.055	1.14	1.01	1.30	0.032	1.08	0.91	1.30	0.38	-0.110	0.003	-0.090	0.289
0.90	1.07	0.657	1.22	1.04	1.43	0.013	1.00	0.69	1.45	0.985	1.02	0.73	1.41	0.924	1.15	0.70	1.89	0.58	-0.050	0.152	0.160	0.068
0.98	1.15	0.134	1.16	1.01	1.33	0.041	1.48	1.05	2.09	0.026	1.21	0.95	1.55	0.128	1.14	0.84	1.59	0.43	-0.180	0.000	-0.090	0.315
1.00	1.20	0.040	0.94	0.81	1.10	0.438	1.48	1.04	2.10	0.029	1.59	1.17	2.17	0.003	1.04	0.69	1.60	0.86	0.097	0.008	_	_
0.92	1.04	0.471	1.34	1.19	1.54	########	0.80	0.66	0.96	0.019	0.83	0.71	0.97	0.018	1.27	0.97	1.73	0.10	-0.373	0.000	_	_
0.91	1.01	0.115	0.95	0.87	1.04	0.296	0.98	0.80	1.19	0.812	0.89	0.76	1.06	0.186	0.97	0.77	1.23	0.81	-0.661	0.000	-0.759	0.000
0.90	1.07	0.637	1.07	0.92	1.24	0.400	1.72	1.13	2.63	0.012	1.42	1.03	1.94	0.032	1.00	0.65	1.51	0.98	0.180	0.000	0.150	0.078
1.03	1.19	0.004	1.11	0.98	1.25	0.102	1.30	0.98	1.73	0.066	1.13	0.91	1.40	0.272	0.89	0.65	1.22	0.48	0.120	0.001	_	_
